# Overexpression of miR-10a-5p facilitates the progression of osteoarthritis

**DOI:** 10.18632/aging.102989

**Published:** 2020-04-13

**Authors:** Hui-Zi Li, Xiang-He Xu, Nan Lin, Da-Wei Wang, Yi-Ming Lin, Zhong-Zhen Su, Hua-Ding Lu

**Affiliations:** 1 Department of Orthopaedics, The Fifth Affiliated Hospital of Sun Yat-sen University, Zhuhai 519000, Guangdong, China; 2 Department of Interventional Medicine, The Fifth Affiliated Hospital Sun Yat-sen University, Zhuhai 519000, Guangdong, China; 3 Guangdong Provincial Key Laboratory of Biomedical Imaging, The Fifth Affiliated Hospital, Sun Yat-sen University, Zhuhai 519000, Guangdong, China; 4 Department of Medical Ultrasonics, The Fifth Affiliated Hospital of Sun Yat-sen University, Zhuhai 519000, Guangdong, China

**Keywords:** osteoarthritis, miR-10a-5p, HOXA3, the whole transcriptome sequencing, integrated bioinformatics analyses

## Abstract

The current study was aimed at exploring the potential roles and possible mechanisms of miR-10a-5p in osteoarthritis (OA). We performed RT-qPCR, Western blot, CCK8, EdU Assay, and flow cytometry assay to clarify the roles of miR-10a-5p in OA. Furthermore, the whole transcriptome sequencing together with integrated bioinformatics analyses were conducted to elucidate the underlying mechanisms of miR-10a-5p involving in OA. Our results demonstrated that miR-10a-5p was upregulated in OA and acted as a significant contributing factor for OA. A large number of circRNAs, lncRNAs, miRNAs, and mRNAs were identified by overexpressing miR-10a-5p. Functional enrichment analyses indicated that these differentially-expressed genes were enriched in some important terms including PPAR signaling pathway, PI3K-Akt signaling pathway, and p53 signaling pathway. A total of 42 hub genes were identified in the protein-protein interaction network including SERPINA1, TTR, APOA1, and A2M. Also, we constructed the network regulatory interactions across coding and noncoding RNAs triggered by miR-10a-5p, which revealed the powerful regulating effects of miR-10a-5p. Moreover, we found that HOXA3 acted as the targeted genes of miR-10a-5p and miR-10a-5p contributed to the progression of OA by suppressing HOXA3 expression. Our findings shed insight on regulatory mechanisms of miR-10a-5p, which might provide novel therapeutic targets for OA.

## INTRODUCTION

Osteoarthritis (OA), a leading cause of joint damage and disability, is associated with impaired quality of life, shortened working duration, and increased all-cause mortality [1–3]. The prevalence of OA continues to grow in recent years, which will further deteriorate with aging population and obesity epidemic [4–6]. A study based on the National Health Interview Survey indicates that approximately 14 million persons have symptomatic knee OA and more than half of them are younger than 65 years old, with high possibility for joint disability over the next 30 years [7]. Several pharmacological agents are helpful in improving the symptoms of early-stage OA, but they can hardly inhibit or block the pathological progression of OA [8]. Eventually, most of advanced-stage victims may undergo total joint replacement owing to serious afflicted joint pain and disability [9]. Mountains of studies suggest that aberrant expression of genes are associated with the initiation and progression of OA [10], but the exact pathogenesis remains to be further clarified.

Non-coding RNAs (ncRNAs) are a group of RNA molecules accounting for a large proportion of the RNA and there are several types of ncRNAs, such as microRNAs (miRNAs), long non-coding RNAs (lncRNAs), and circular RNAs (circRNAs) [11, 12]. miRNA, one of the most widely-studied ncRNAs, suppresses the expression of target genes through binding to the 3’UTR of their corresponding mRNAs [13]. LncRNAs are a group of ncRNAs with lengths exceeding 200 nucleotides and can regulate gene expression at transcription and post-transcription level [14]. CircRNAs are a special type of ncRNAs with covalently closed ring structure originating from back-splicing of pre-mRNAs at the downstream 5' splice site and the 3' splice site [15]. The rapid development of RNA-sequencing and bioinformatics analysis reveal that numerous miRNAs, lncRNAs and circRNAs are identified in various human diseases, including OA [16–18]. Accumulating evidence indicated that complicated communication networks across mRNA, miRNAs, lncRNAs and circRNAs participated in the onset and progression of OA and acted as potential therapeutic targets for OA. Wang and coworkers found that miR-483-5p was upregulated in OA and inhibition of miR-483-5p attenuated the progression of OA via Matn3 and Timp2 *in vivo* [19]. LncRNA-TM1P3 was significantly over-expressed in OA and promoted extracellular matrix degradation by regulating miR-22/TGF-β signaling/MMP13 pathway [20]. CircSERPINE2 participated in regulating chondrocytes metabolism and apoptosis through miR-1271/ERG axis and overexpression of CircSERPINE2 repressed the progression of OA in the rabbit model [17]. Actually, the crosstalk across coding and noncoding RNAs has been attributed increasing importance since Salmena et al. firstly proposed the ceRNA (competing endogenous RNA) hypothesis in 2011 [21]. Of the ceRNA networks, miRNAs play a central role to connect coding and noncoding RNAs via microRNA response elements (MREs), which act as the critical communicate bridges [21]. Furthermore, increasing studies suggested that miRNAs participated in the development and progression of diseases through triggering alterations of the whole transcriptome. For instance, overexpression of circRNA-Filip1l induced by decrease of miRNA-1224 facilitated chronic inflammatory pain via upregulation of Ubr5 in an Ago2-dependent manner [22]. Ye and colleagues also revealed that overexpression of miR-145 suppressed breast cancer progression and induced alterations of mRNAs, miRNAs, lncRNAs and circRNAs [23]. Therefore, it is essential to further explore the network regulatory interactions across coding and noncoding RNAs induced by miRNAs, which may provide novel diagnostic biomarkers and potential drug targets for OA. miR-10a-5p is a member of miR-10 family and could regulate cell proliferation, apoptosis, and inflammatory factors in many inflammation-associated diseases, including atopic dermatitis and rheumatoid arthritis [24–26]. Previous studies also indicated that miR-10a-5p was over-expressed in OA [16, 27]. However, the potential roles and molecule mechanisms of miR-10a-5p in OA were not fully elucidated.

**Figure 1:**
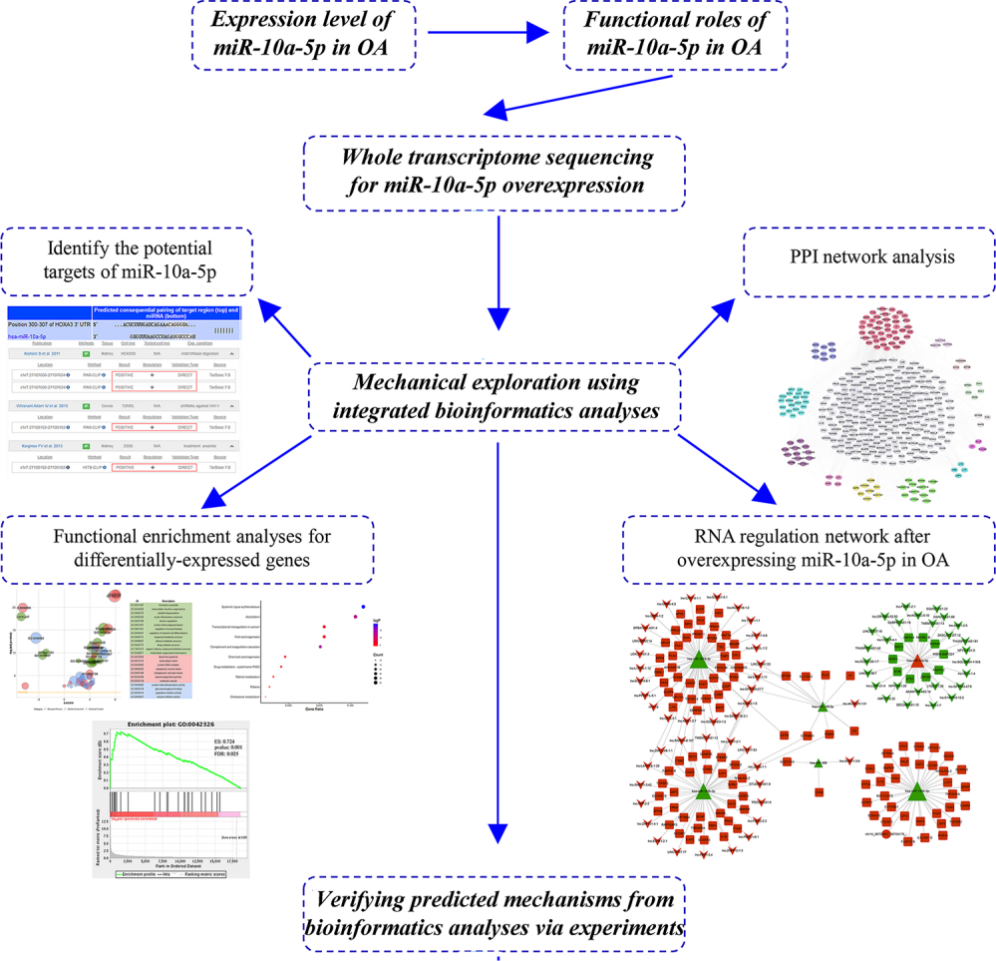
**The workflow of study design.**

In the current study, we verified that miR-10a-5p was significantly upregulated in OA and acted as a potential promising biomarker. Furthermore, we found that miR-10a-5p inhibited chondrocyte proliferation and promoted chondrocyte apoptosis. To in-depth explore the mechanisms of miR-10a-5p in OA, we performed RNA sequence for the whole transcriptome. Subsequently, integrated bioinformatics analyses were employed to illuminate the alterations of the whole transcriptome induced by miR-10a-5p. The workflow of study design is shown in Figure 1. Our findings may open up a new sight into regulatory mechanism of miRNAs, which may provide novel therapeutic targets for OA.

## RESULTS

### miR-10a-5p is upregulated in OA and acts as a potential promising biomarker

**Figure 2:**
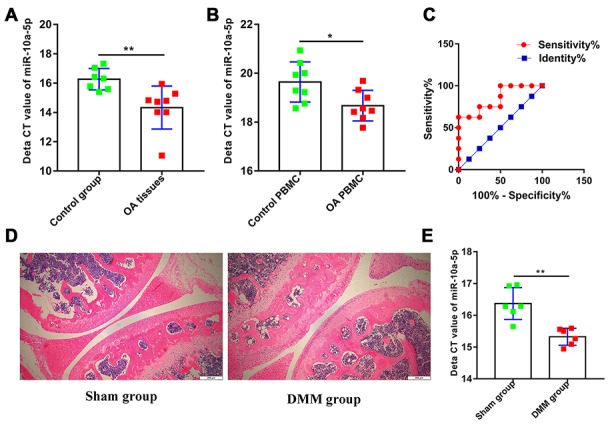
**miR-10a-5p is upregulated in OA and acts as a potential promising biomarker.** (**A**) The relative expression of miR-10a-5p in OA and control cartilage tissues analyzed by RT-qPCR (8 OA cartilage vs. 7 control cartilage, **P < 0.01); (**B**) The relative expression of miR-10a-5p in OA and control PBMC analyzed by RT-qPCR (8 OA cartilage vs. 8 control cartilage, *P < 0.05); (**C**) ROC analysis of miR-10a-5p in PBMC for the diagnosis of OA (AUC,0.84; P=0.02); Representative pictures of articular cartilage in sham group (**D** left) and DMM group (**D** right) stained by H&E. Scale bar, 200 um. (**E**) The relative expression of miR-10a-5p in sham group and DMM group analyzed by RT-qPCR (6 sham vs. 6 DMM cartilage, **P < 0.01).

**Figure 3:**
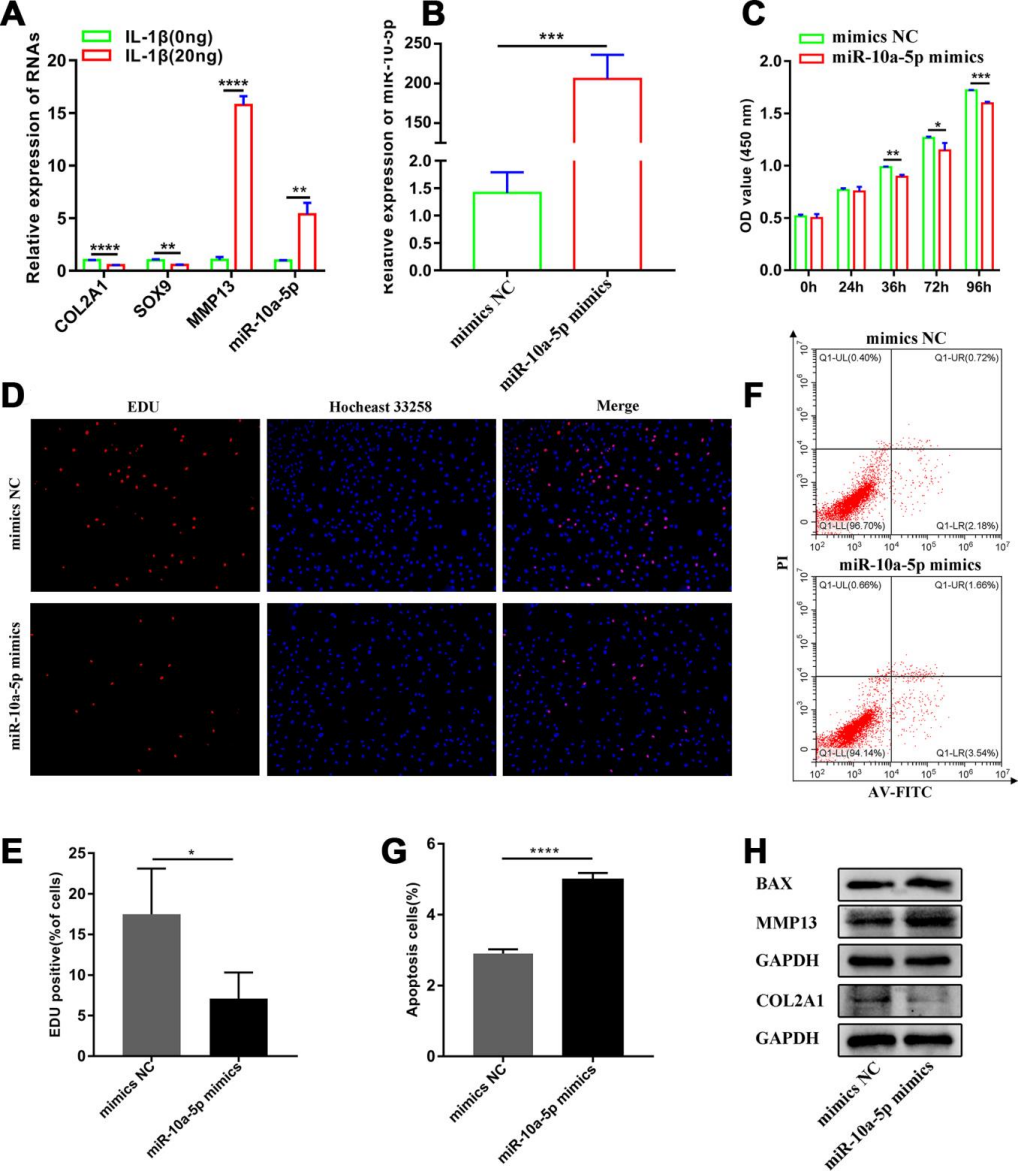
**miR-10a-5p acts as a significant contributing factor for OA.** (**A**) The relative expression of COL2A1, MMP13, SOX9, and miR-10a-5p in control and IL-1β induced Hc-a analyzed by RT-qPCR (n=3; **P < 0.01, ****P < 0.0001). (**B**) The relative expression of miR-10a-5p after transfecting miR-10a-5p mimics, ***P < 0.001. (**C**) The effect of miR-10a-5p overexpression on cell proliferation detected by CCK8 assay (n=3; *P < 0.05, **P < 0.01, ***P < 0.001). (**D**, **E**) The effect of miR-10a-5p overexpression on cell proliferation detected by EDU assay (n=3; *P < 0.05). (**F**, **G**) The effect of miR-10a-5p overexpression on cell apoptosis detected by flow cytometry assay (n=3; ****P < 0.0001). (**H**) Effects of miR-10a-5p overexpression on Col2a1, MMP13, BAX, and GAPDH protein levels detected by western blot.

To explore the potential roles of miR-10a-5p in OA, we firstly detected the relative expression level of miR-10a-5p in OA and normal articular cartilage using RT-qPCR (Figure 2A). The results indicated that miR-10a-5p was upregulated in OA articular cartilage. Previous studies indicated that miRNAs in PBMC acted as promising biomarkers [28, 29]. In the current study, we also explore whether miR-10a-5p can act as a potential biomarker in OA. The results showed that miR-10a-5p was upregulated in PBMC of OA patients and might act as a promising predictor for OA with an area under the curve of 0.84 (95% confidence interval 0.65–1.04, P=0.02; Figure 2B–2C). Consistently, we also verified that miR-10a-5p was significantly over-expressed in mouse OA model (Figure 2D–2E) and IL-1β induced chondrocytes (Figure 3A).

### Overexpression of miR-10a-5p inhibits chondrocyte proliferation and promotes chondrocyte apoptosis and cartilage matrix degradation

Subsequently, we evaluated the effect of miR-10a-5p on chondrocyte proliferation, apoptosis and metabolism after overexpressing miR-10a-5p in HC-a (Figure 3B). CCK-8 assay, EDU assay, and flow cytometry assay indicated that overexpression of miR-10a-5p inhibited chondrocyte proliferation (Figure 3C–3E) and promoted chondrocyte apoptosis (Figure 3F–3G). Also, we performed western blot to explore the effect of miR-10a-5p on extracellular matrix metabolism. Western blot results indicated that overexpression of miR-10a-5p inhibited the expression of COL2A1 and promoted the expression of MMP13 (Figure 3H). Taken together, these results demonstrated that miR-10a-5p may act as a significant contributing factor for OA.

### Overexpression of miR-10a-5p induces alteration of the whole transcriptome in human primary chondrocyte

**Figure 4:**
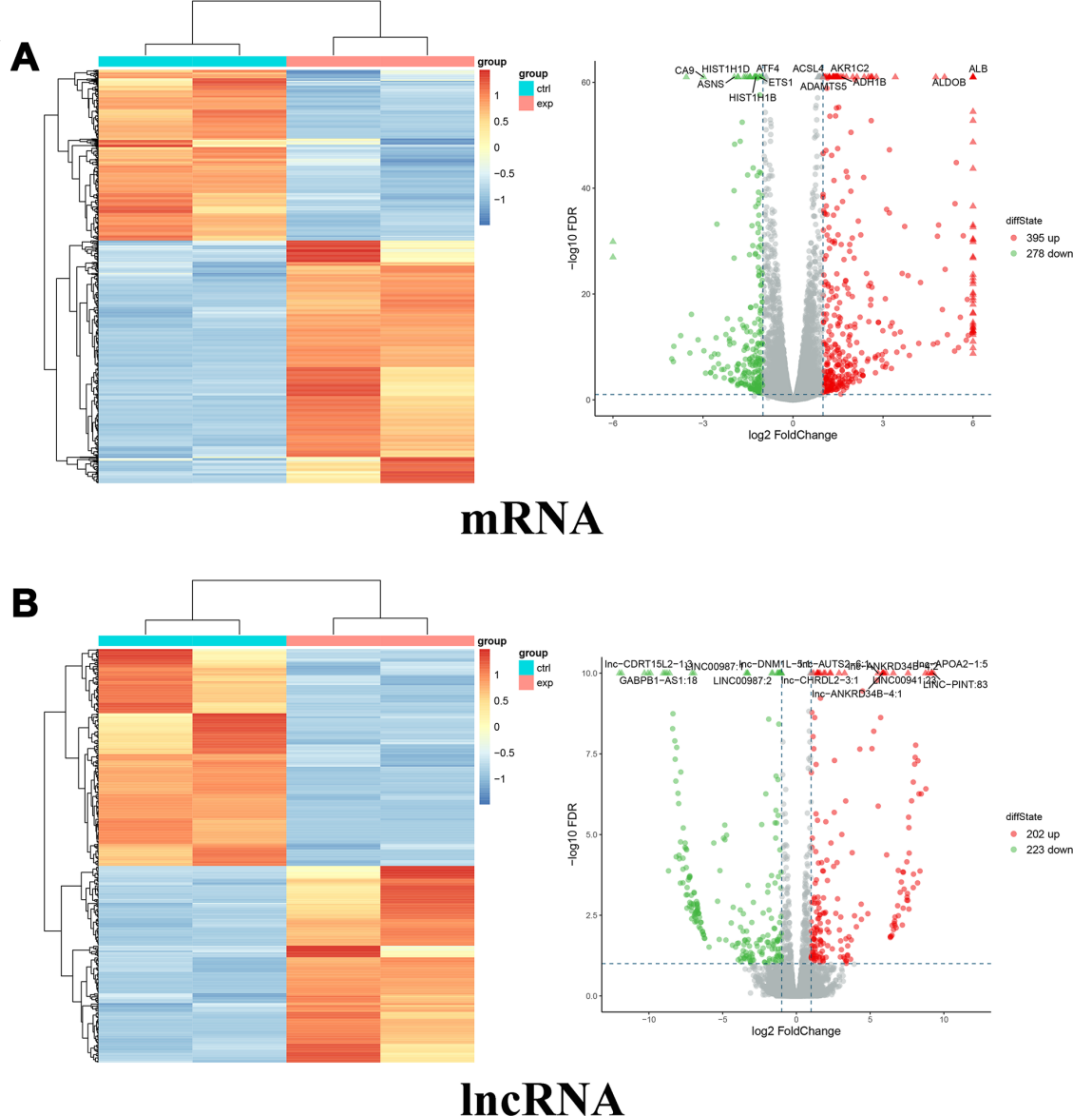
**Differentially expressed mRNAs and lncRNAs between miR-10a-5p overexpression group (exp) and control group (ctrl).** (**A**) Heat map (left) and volcano plot (right) of differentially expressed mRNAs (red color denotes upregulated mRNAs and green color denotes downregulated mRNAs). (**B**) Heat map (left) and volcano plot (right) of differentially expressed lncRNAs (red color denotes upregulated lncRNAs and green color denotes downregulated lncRNAs).

**Figure 5:**
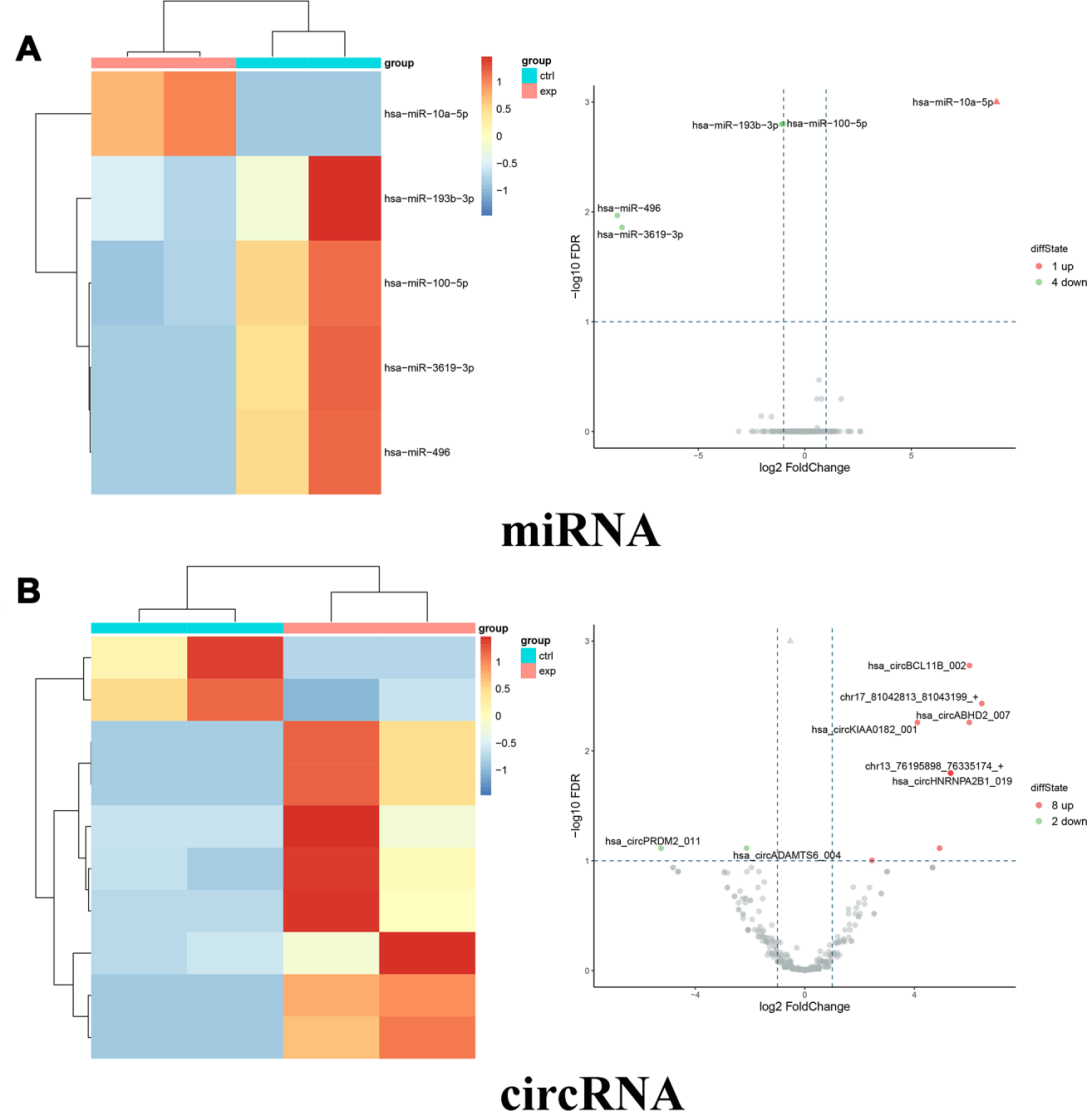
**Differentially expressed miRNAs and circRNAs between miR-10a-5p overexpression group (exp) and control group (ctrl).** (**A**) Heat map (left) and volcano plot (right) of differentially expressed miRNAs (red color denotes upregulated miRNAs and green color denotes downregulated miRNAs). (**B**) Heat map (left) and volcano plot (right) of differentially expressed circRNAs (red color denotes upregulated circRNAs and green color denotes downregulated circRNAs).

**Figure 6:**
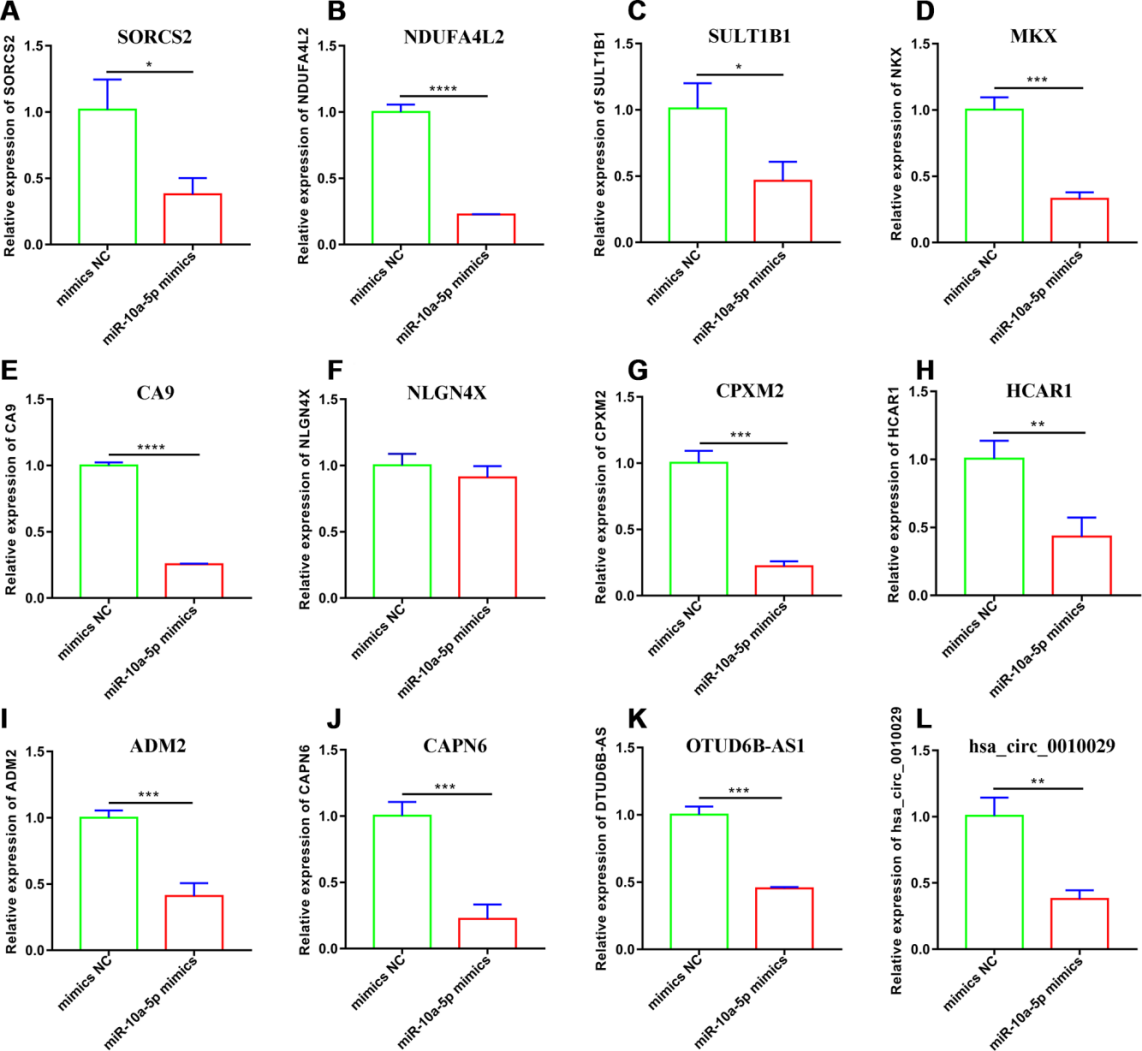
**Validation of 12 selected downregulated genes after miR-10a-5p overexpression through RT-qPCR.** (**A**) SORCS2; (**B**) NDUFA4L2; (**C**) SULT1B1; (**D**) MKX; (**E**) CA9; (**F**) NLGN4X; (**G**) CPXM2; (**H**) HCAR1; (**I**) ADM2; (**J**) CAPN6; (**K**) OTUD6B-AS1; (**L**) hsa_circ_0010029.(n=3; *P < 0.05, **P < 0.01, ***P < 0.001, ****P < 0.0001).

To explore the potential mechanism of miR-10a-5p in OA, we performed the whole transcriptome sequencing to identify differentially-expressed genes on HC-a via miR-10a-5p overexpressing. These results indicated that overexpression of miR-10a-5p induced the alteration of the whole transcriptome, which involved 395 up-regulated and 278 down-regulated mRNAs (Figure 4A), 202 up-regulated and 223 down-regulated lncRNAs (Figure 4B), 1 up-regulated and 4 down-regulated miRNAs (Figure 5A), and 8 up-regulated and 2 down-regulated circRNAs (Figure 5B). The detailed differentially-expressed mRNAs, lncRNAs, miRNAs, and circRNAs were showed in Supplementary Tables 2–5. Moreover, RT-qPCR showed that the expression level of 12 selected differentially-expressed genes were basically consistent with the results of RNA-seq (Figure 6).

### Functional enrichment analyses for differentially-expressed mRNAs, circRNAs, and lncRNAs

**Figure 7:**
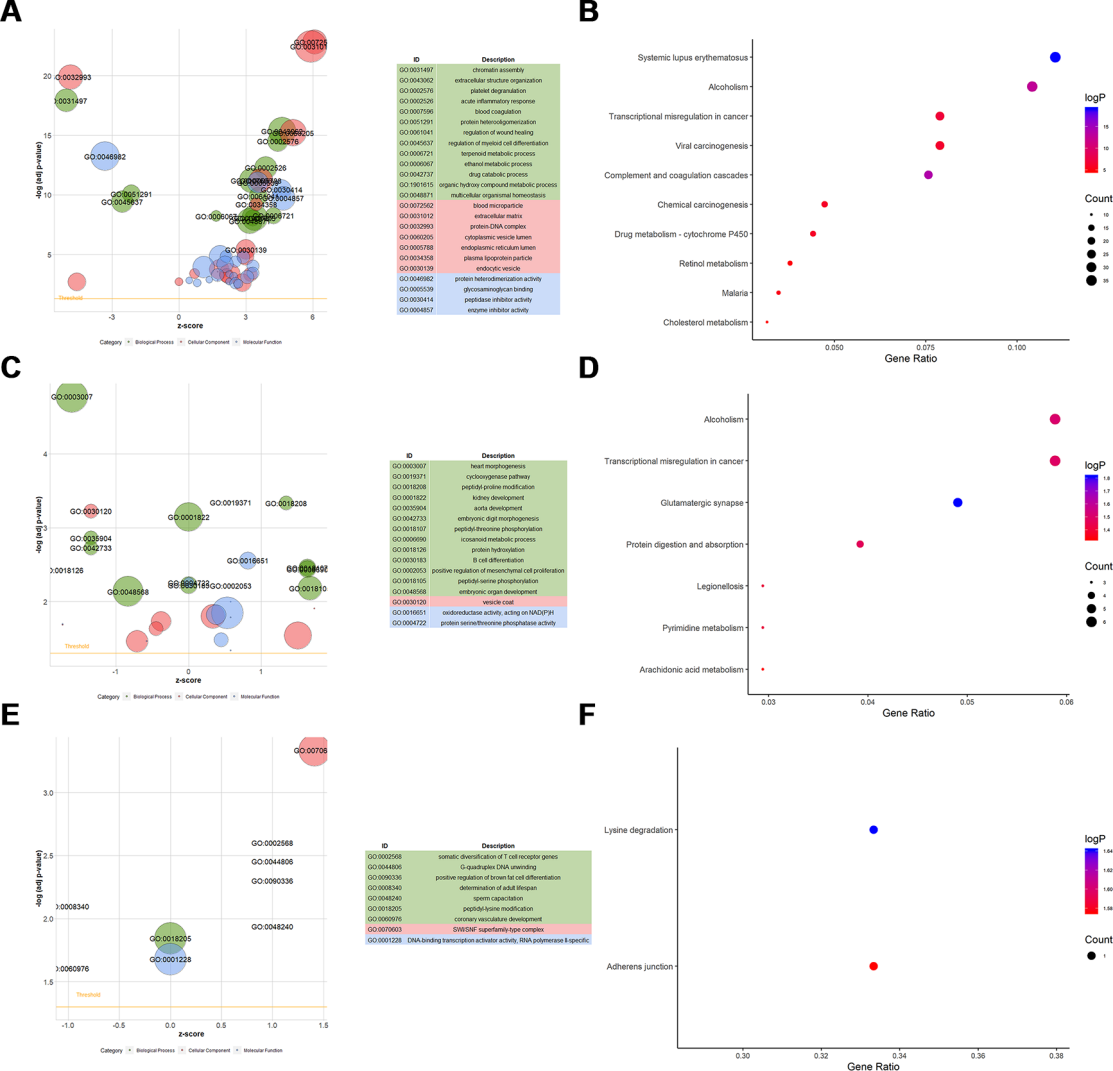
**Functional enrichment analyses for differentially-expressed mRNAs, lncRNAs, and circRNAs.** (**A**) GOBubble plot shows GO terms enriched in differentially-expressed mRNAs. (**B**) KEGG terms enriched in differentially-expressed mRNAs. (**C**) GOBubble plot shows GO terms enriched in *ci*-genes of differentially-expressed lncRNAs. (**D**) KEGG terms enriched in *ci*-genes of differentially-expressed lncRNAs. (**E**) GOBubble plot shows GO terms enriched in parent genes of differentially-expressed circRNAs. (**F**) KEGG terms enriched in parent genes of differentially-expressed circRNAs.

**Figure 8:**
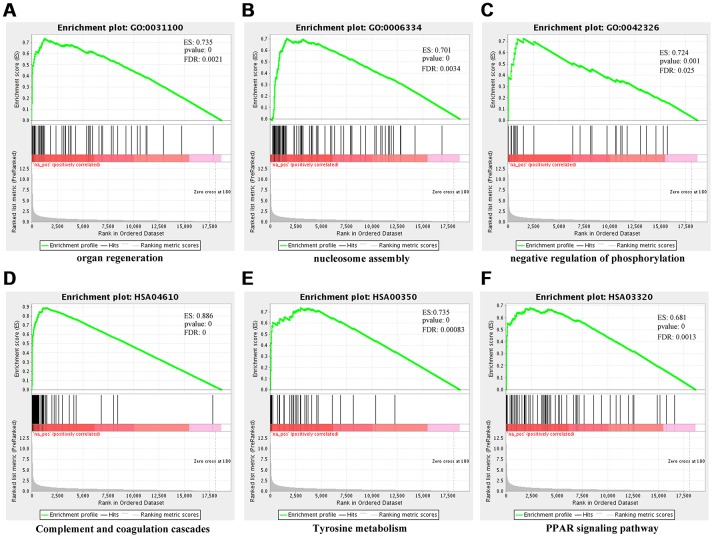
**Gene set enrichment analysis (GSEA) for differentially-expressed mRNAs using NGSEA.** Representative three significantly GO enrichment plot in GSEA: (**A**) organ regeneration (GO:0031100); (**B**) nucleosome assembly (GO:0006334); (**C**) negative regulation of phosphorylation. Representative three significantly KEGG enrichment plot in GSEA: (**D**) complement and coagulation cascades (HSA04610); (**E**) tyrosine metabolism (HSA00350); (**F**) PPAR signaling pathway (HSA03320).

Furthermore, we undertook functional enrichment analyses for these dysregulated genes. GO enrichment analysis for dysfunctional mRNAs indicated that they were mainly enriched in chromatin assembly (GO:0031497), extracellular structure organization (GO:0043062), protein-DNA Complex (GO:0032993), extracellular matrix (GO:0031012), and glycosaminoglycan binding (GO:0005539) (Figure 7A, Supplementary Tables 6–8). KEGG enrichment analysis for dysfunctional mRNAs revealed that they mainly enriched in systemic lupus erythematosus (HSA05322), alcoholism (HSA05034), complement and coagulation cascades (HSA04610), and cholesterol metabolism (HSA04979) (Figure 7B, Supplementary Table 9). Functional enrichment analyses for *cis-genes* of differentially expressed lncRNAs showed that they were enriched in positive regulation of mesenchymal cell proliferation (GO:0002053), peptidyl-proline modification (GO:0018208), Alcoholism (HSA05034), Arachidonic acid metabolism (HSA00590) (Figure 7C, 7D, Supplementary Tables 10–13). Functional enrichment analyses for parental genes of differentially expressed circRNAs showed that they mainly participated in somatic diversification of T cell receptor genes (GO:0002568), positive regulation of brown fat cell differentiation (GO:0090336), Adherens junction (HSA04520) (Figure 7E, 7F, Supplementary Tables 14–17). Also, GSEA for differentially expressed mRNAs verified that they were mainly associated with organ regeneration (GO:0031100), nucleosome assembly (GO:0006334), negative regulation of phosphorylation (GO:0042326), complement and coagulation cascades (HSA04610), tyrosine metabolism (HSA00350), PPAR signaling pathway (HSA03320), PI3K-Akt signaling pathway (HSA04151), p53 signaling pathway (HSA04115), FoxO signaling pathway (HSA04068) (Figure 8A–8F, Supplementary Tables 18–19).

### PPI network analysis

**Figure 9:**
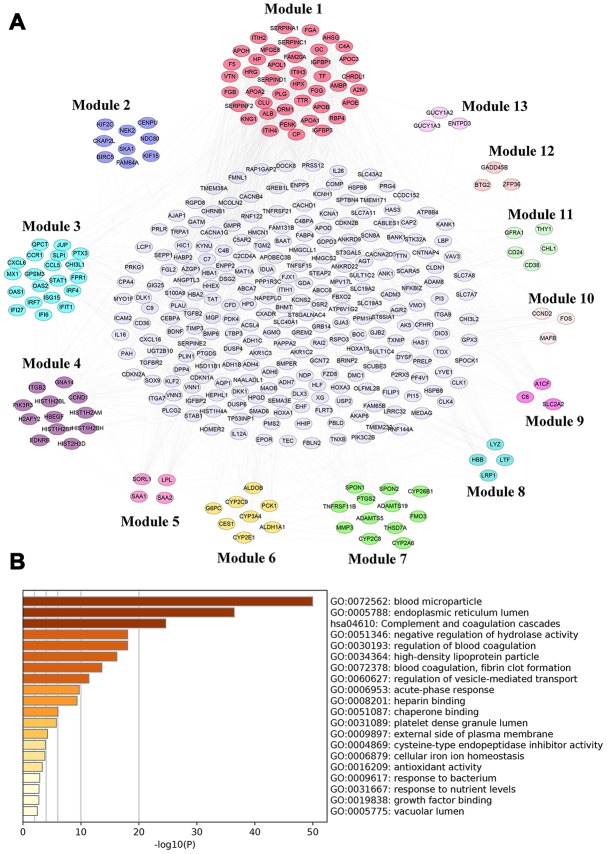
**PPI network analysis for differentially-expressed mRNAs.** (**A**) PPI network of differentially-expressed mRNAs analyzed by STRING database and Module (1-13) were selected from PPI network using MCODE analysis. (**B**) Functional enrichment analysis of hub genes (Module 1) were performed using Metascape.

The protein-protein regulatory network contained 365 nodes and 2210 edges (Figure 9A). MCODE analysis indicated that thirteen modules were extract from the PPI network, but the 42 genes from module 1 had the highest node degree according to CytoHubba (Supplementary Table 20). Therefore, these genes from module 1 were identified as hub genes. Subsequently, we conducted functional enrichment analyses for these hub genes using Metascape [30]. The results revealed that they were mainly enriched in blood microparticle (GO:0072562), endoplasmic reticulum lumen (GO:0005788), complement and coagulation cascades (HSA04610) (Figure 9B).

### Construction of ceRNA regulatory networks

**Figure 10:**
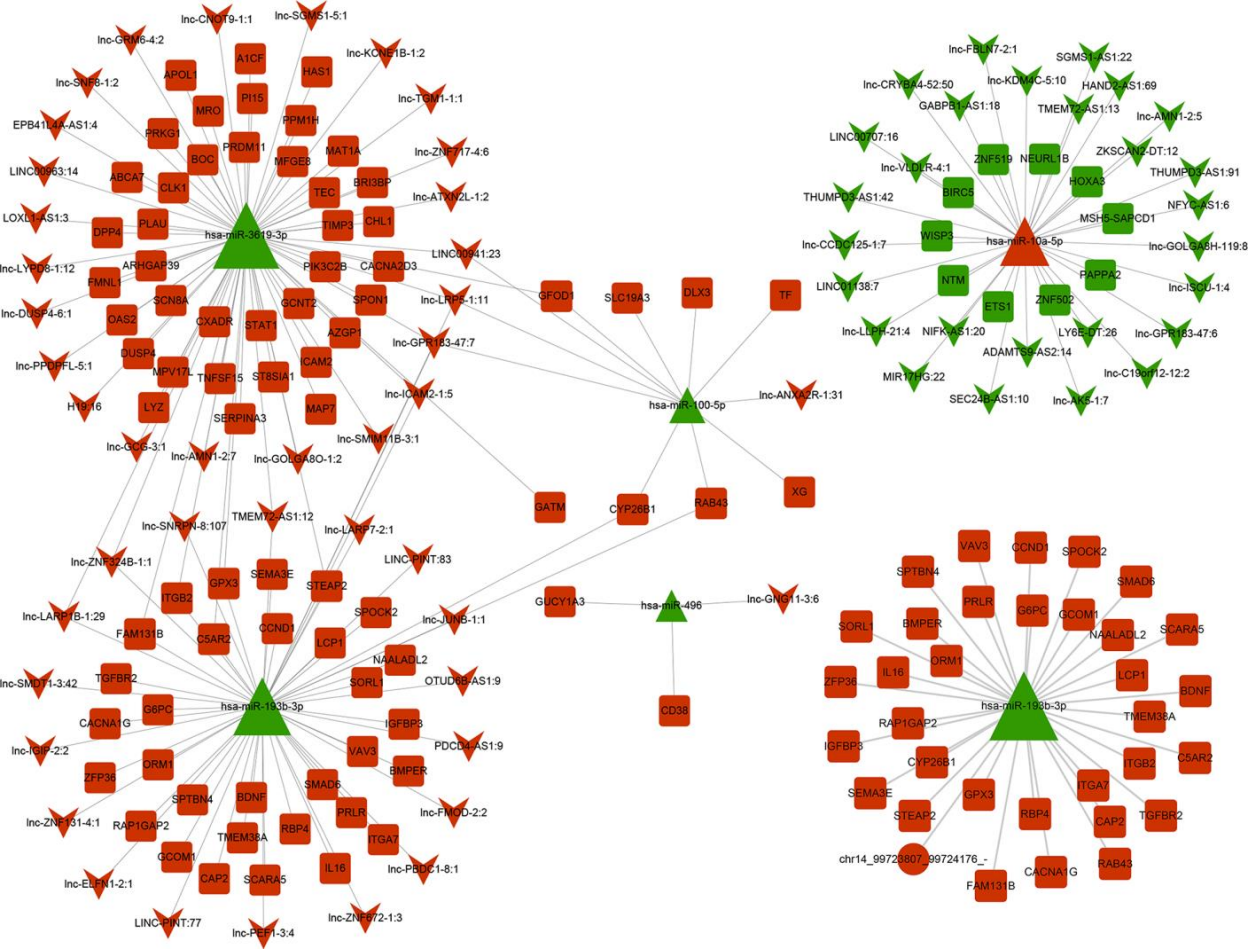
**ceRNA regulation network centered on downregulated and upregulated miRNAs after overexpressing miR-10a-5p in Hc-a.** The circle represents circRNA, square represents mRNA and triangle represents miRNA, and inverted triangle represents lncRNA.

We also constructed the network regulatory interactions across coding and noncoding RNAs triggered by miR-10a-5p. Considering that miRNAs play critical pivotal roles in ceRNA interactions, we constructed ceRNA regulatory networks centralized on significant upregulated and downregulated miRNAs. Downregulated miRNAs-associated ceRNA networks contained 1 circRNA, 42 lncRNA, 5 miRNA and 112 mRNA (Figure 10). Especially, miR-10a-5p was the only upregulated miRNA after miR-10a-5p overexpression. The ceRNA network centered on miR-10a-5p consisted of 27 lncRNA, 1 miRNA, and 10 mRNA (Figure 10). The complicated regulatory networks across coding and noncoding RNAs revealed the powerful regulating effects of miR-10a-5p.

### HOXA3 acts as a targeted gene of miR-10a-5p

**Figure 11:**
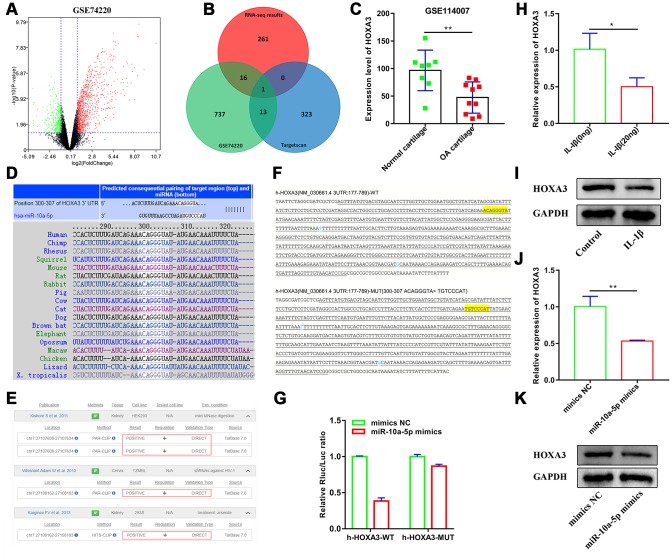
**HOXA3 acts as a targeted gene of miR-10a-5p.** (**A**) Volcano plot of differentially expressed mRNAs between control group and IL-1β induced primary chondrocyte group from GSE74220. (**B**) Venn plot of the overlapping mRNA across the downregulated mRNAs in GSE74220, the predicted targets of miR-10a-5p in Targetscan, and the downregulated mRNAs in our RNA-seq. (**C**) The expression level of HOXA3 in OA cartilage from GSE114007, **P < 0.01. (**D**) Targetscan shows that the seed region of miR-10a-5p is well-paired with 3’UTR of HOXA3, which was conservative among vertebrates. (**E**) High-throughput experiments verified that HOXA3 was the direct target of miR-10a-5p in HEK293, TZMBL, and 293S from TarBase v.8. (**F**) The sequencing results of cloned fragments in luciferase reporter vectors and the yellow highlighted sequence is the target test site. (**G**) Interaction between miR-10a-5p and HOXA3 was verified by luciferase report assay in 293T cells. (**H**, **I**) The relative expression of HOXA3 in control and IL-1β induced Hc-a analyzed by RT-qPCR and western blot (n=3, *P < 0.05). (**J**, **K**) The relative expression of HOXA3 after transfecting miR-10a-5p mimics analyzed by RT-qPCR and western blot (n=3, **P < 0.01).

To seek for the potential target of miR-10a-5p, we firstly identify the downregulated mRNAs in OA. A total of 767 downregulated mRNAs were identified in IL-1β induced primary chondrocytes after processing the data of GSE74220 using BioJupies [31] (Figure 10A). Furthermore, we investigated that HOXA3 was the only overlapping mRNA across the downregulated mRNAs in GSE74220, the predicted targets of miR-10a-5p via Targetscan, and the downregulated mRNAs in our RNA-seq results (Figure 11B). Subsequently, we found that HOXA3 was downregulated in OA cartilage after analyzing the results of GSE114007 (Figure 11C). The results of Targetscan showed that the seed region of miR-10a-5p was well-paired with 3’UTR of HOXA3, which was conservative among vertebrates (Figure 11D). According to TarBase v.8 [32], high-throughput experiments including PAR-CLIP and HITS-CLIP indicated that miR-10a-5p was the direct inhibition to HOXA3 in HEK293, TZMBL, and 293S (Figure 11E). Furthermore, dual luciferase reporter assay confirmed that luciferase activity of HOXA3-wt was obviously inhibited by miR-10a-5p mimics as compared with that in HOXA3-mut group (Figure 11F–11G). RT-qPCR and western blot results showed that HOXA3 was significantly inhibited in IL-1β induced HC-a (Figure 11H–11I) and miR-10a-5p overexpression (Figure 11J–11K). Taken together, these results indicated that HOXA3 was downregulated in OA and acted as the targeted genes of miR-10a-5p.

### miR-10a-5p exerts biological functions in OA cell model by targeting HOXA3

**Figure 12:**
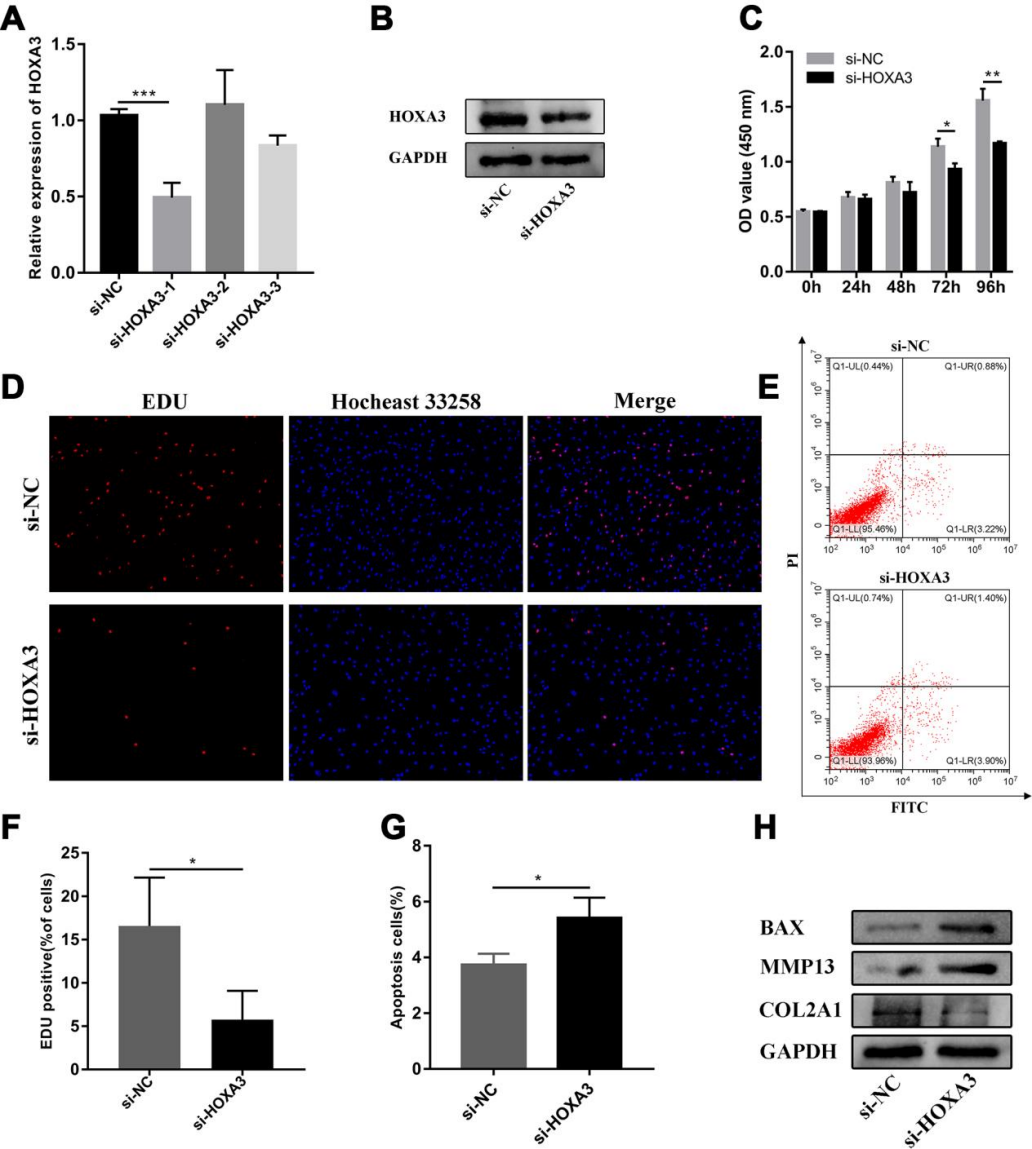
**Silence of HOXA3 inhibited chondrocyte proliferation and promoted chondrocyte apoptosis.** (**A**) The relative expression of HOXA3 after transfecting si-HOXA3 analyzed by RT-qPCR (n=3; ***P < 0.001). (**B**) The relative expression of HOXA3 after transfecting si-HOXA3 analyzed by western blot. (**C**) The effect of HOXA3 knockdown on cell proliferation detected by CCK8 assay (n=3; *P < 0.05, **P < 0.01). (**D**, **F**) The effect of HOXA3 knockdown on cell proliferation detected by EDU assay (n=3; *P < 0.05). (**E**, **G**) The effect of HOXA3 knockdown on cell apoptosis detected by flow cytometry assay (n=3; *P < 0.05). (**H**) Effects of HOXA3 knockdown on Col2a1, MMP13, BAX, and GAPDH protein levels detected by western blot.

**Figure 13:**
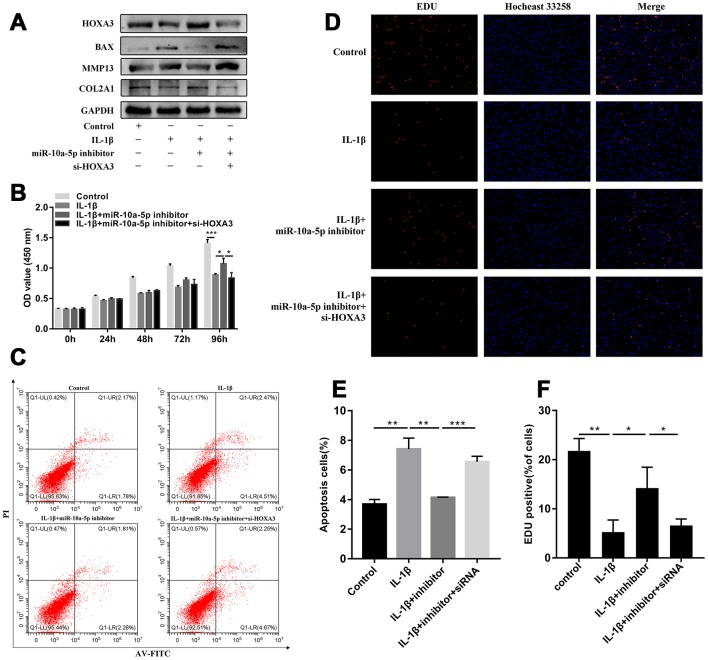
**miR-10a-5p functioned in IL-1β induced Hc-a through targeting HOXA3.** Hc-a was treated with IL-1β and then co-transfected miR-10a-5p inhibitor and si-HOXA3. (**A**) HOXA3, Col2a1, MMP13, BAX, and GAPDH protein levels were detected by western blot. (**B**, **D**, **F**) cell proliferation detected by CCK8 (**B**; n=3; *P < 0.05, ***P < 0.001) and EDU assay (**D**, **F**; n=3, *P < 0.05, **P < 0.01, ***P < 0.001). (**C**, **E**) cell apoptosis detected by flow cytometry assay (n=3, **P < 0.01, ***P < 0.001).

Based on the above results, we found that miR-10a-5p was a significant contributor to the pathogenesis of OA. RNA-sequencing together with integrated bioinformatics analyses identified that miR-10a-5p promoted the progression of OA through triggering the alterations of the whole transcriptome. Subsequently, one of the most representative downstream genes, HOXA3 (the targeted gene of miR-10a-5p) was chosen to verify the predicted mechanisms from bioinformatics analyses. Three siRNAs were designed to knockdown the expression of HOXA3. RT-qPCR results showed that si-HOXA3-1 had the highest silence efficiency, which was further confirmed by western blot (Figure 12A, 12B). Therefore, si-HOXA3-1 was chosen to perform subsequent experiments. CCK-8 assay, EDU assay, flow cytometry assay, and western blot verified that silence of HOXA3 significantly inhibited chondrocyte proliferation (Figure 12C, 12D, 12F), promoted chondrocyte apoptosis (Figure 12E, 12G) and cartilage matrix degradation (Figure 12H). Next, we further investigated whether miR-10a-5p functioned in IL-1β induced HC-a through targeting HOXA3. We treated HC-a with IL-1β and then co-transfected miR-10a-5p inhibitor and si-HOXA3. Western blot also indicated that si-HOXA3 partly reversed the protective effect of miR-10a-5p inhibitor on IL-1β treated HC-a (Figure 13A). CCK-8 and EDU assay indicated that miR-10a-5p inhibitor partly restored IL-1β-induced inhibition of HC-a proliferation, while si-HOXA3 attenuated the effect of miR-10a-5p inhibitor on IL-1β treated HC-a (Figure 13B, 13D, 13F). Flow cytometry assay showed that miR-10a-5p inhibitor partly reversed IL-1β-induced chondrocyte apoptosis, while si-HOXA3 antagonized the effect of miR-10a-5p inhibitor on IL-1β treated chondrocyte (Figure 13C, 13E). Collectively, these finding indicated that miR-10a-5p exerts biological functions in OA cell model by targeting HOXA3.

## DISCUSSION

In the study, our findings indicated that miR-10a-5p was upregulated in OA and acted as a potential biomarker. Moreover, we found that miR-10a-5p overexpression inhibited chondrocyte proliferation and facilitated chondrocyte apoptosis and cartilage matrix degradation. Subsequently, RNA-seq together with integrated bioinformatics analyses was performed to comprehensively explore the potential mechanisms of miR-10a-5p in OA. Further experiments based on bioinformatics analyses demonstrated that miR-10a-5p exerted biological functions in OA cell model by targeting HOXA3.

The results of RNA-seq revealed that overexpression of miR-10a-5p triggered the alteration of mRNAs, miRNAs, lncRNAs, and circRNAs. Accordingly, functional enrichment analyses including GO, KEGG, and GSEA were performed for these differentially-expressed genes induced by miR-10a-5p. GO analyses indicated that they were enriched in many significant terms, such as extracellular structure organization, extracellular matrix, and glycosaminoglycan binding. It was well-known that extracellular matrix was the main component of articular cartilage and extracellular matrix degradation was regarded as the key pathological hallmark of OA. Numerous studies revealed that many promising therapeutic targets aimed at promoting extracellular matrix generation significantly repressed the progression of OA [33, 34]. Also, glycosaminoglycans (GAGs) were the important building foundation of articular cartilage and GAGs bindings may play important roles in pathogenesis of OA. Flannery and coworkers identified that ADAMTS-4 existed multiple GAG-binding sites and may contribute to extracellular matrix degradation through recognizing these bindings in the aggrecan core protein [35]. Another study indicated that PRELP, a GAG binding protein, inhibited osteoclastogenesis through repressing NF-κB transcriptional activity [36]. Considering that PRELP was highly-expressed in cartilage and activation of NF-κB signaling pathway was a significant contributor in OA [36, 37], the potential roles of PRELP in OA deserved further exploration. KEGG analyses indicated that these differentially-expressed genes induced by miR-10a-5p were enriched in PPAR signaling pathway, PI3K-Akt signaling pathway, and P53 signaling pathway. PPARs used to be regarded as a group of ligand-inducible transcription factors participating in lipid and glucose homeostasis, but increasing evidence showed that they remained fundamental to normal cartilage development [38]. Further studies indicated that PPARγ deficiency facilitated the formation of accelerated spontaneous OA, whereas PPARγ preservation alleviated the development and progression of OA [39, 40]. Furthermore, increasing evidence indicated that PI3K-Akt signaling pathway participated in the pathogenesis of OA and inhibiting PI3K/AKT/NF-κB signal pathway repressed the progression of osteoarthritis [41, 42]. P53 was identified as an ultimate tumor-suppressor gene, but recent studies showed that it also involved in OA chondrocytes apoptosis. Islam and colleagues demonstrated that hydrostatic pressure induced OA chondrocytes apoptosis though upregulating the expression of p53 [43]. Hashimoto and coworkers found that p53 was overexpressed in OA chondrocytes and silencing of p53 can alleviate chondrocytes apoptosis induced by shear strain [44]. These studies implied that downregulation of p53 may act as a potential therapeutic approach in OA treatment. Taken together, these important biological processes and pathways induced by miR-10a-5p played important roles in OA, which enlarged our understanding for the underlying mechanism of miR-10a-5p in OA and may provide novel therapeutic targets for OA.

We also performed PPI network analysis for differentially-expressed mRNAs induced by miR-10a-5p. A total of 42 hub genes were identified in the PPI network including SERPINA1, TTR, APOA1, and A2M. Previous studies found that SERPINA1, a chondrogenic differentiation gene, was significantly up-regulated in OA [45, 46]. Furthermore, Yoshida et al. identified that alpha1-antitrypsin, a protein encoded by SERPINA1, could interact with ADAMTS-4 *in vivo*, although the potential significance of their interaction was largely unknown [47]. Therefore, the possible roles and mechanism of SERPINA1 in OA should be further elucidated. TTR, a common amyloidogenic protein, was found to be highly deposited in OA cartilage and promoted OA progression [48, 49]. Moreover, TTR deposition inducing extracellular matrix degeneration can be alleviated in part by TLR4 and p38 MAPK [48]. APOA1, a protein related to lipid metabolism, was also deposited in OA cartilage [50]. Further studies showed that the expression levels of IL-6, MMP-1 and MMP-3 were significantly upregulated after stimulating chondrocytes and fibroblast-like synoviocytes with ApoA1. Interestingly, Tsezou et al. revealed that cholesterol efflux genes including ApoA1 were significantly downregulated in OA cartilage and activation of cholesterol efflux through LXR agonist increased the expression of ApoA1 [51]. Regardless of those inconsistences, the dysfunction of ApoA1 may act as a critical player in OA and further studies should be warranted to clarify its roles in OA. A2M was identified as an endogenous inhibitor to the ADAMTS family including ADAMTS-4, ADAMTS-5, ADAMTS-7, and ADAMTS-12, which were critical aggrecanase responsible for aggrecan degradation in OA [52, 53]. In addition, intra-articular injection of A2M significantly suppressed the progression of OA *in vivo*, which suggested that A2M may be a promising target for OA treatment [54]. Considering the potential roles of these hub genes in OA, we postulated that miR-10a-5p may promote the development of OA through regulating the important hub genes.

Apart from downregulation of mRNAs, miR-10a-5p overexpression also induced downregulation or upregulation of numerous ncRNAs including miRNAs, lncRNAs, and circRNAs. Many studies revealed that TFs regulated the expression of RNAs. Therefore, downregulation of TFs induced by miR-10a-5p overexpression may be responsible for inhibitory expression of ncRNAs. Conventionally, miRNAs were thought to function in RNA silencing, but recent studies demonstrated that miRNAs located in the nucleus, or NamiRNA could activate gene transcription by targeting enhancers [55]. Accordingly, miR-10a-5p in the nucleus might bind to some key enhancers, thus promoting the upregulation of ncRNAs and mRNAs. We also constructed the ceRNA regulatory networks centralized on significant upregulated and downregulated miRNAs triggered by miR-10a-5p. The regulatory networks reflected the complicated molecular mechanisms of miR-10a-5p involving in OA, which might provide potential scientific foundation for future studies.

Mountains of studies demonstrated that miRNAs regulated the expression of genes through binding to the 3' untranslated regions (UTRs) of target mRNAs, so we also identified the potential direct targets of miR-10a-5p in OA. We found that HOXA3 acted as a downstream target of miR-10a-5p. HOXA3 was a member of homeobox (HOX) genes and previous studies revealed that dysfunction of HOX genes may be associated with initiation and development of OA, although the exact mechanism remained to be further explored [56]. To further verify the results from bioinformatics analyses, we merely chose one of the most representative downstream genes, HOXA3(the targeted gene of miR-10a-5p) to verify the predicted mechanisms. We found that HOXA3 was down-regulated in OA and silencing of HOXA3 inhibited chondrocyte proliferation, promoted chondrocyte apoptosis and cartilage matrix degradation. Furthermore, we also demonstrated that miR-10a-5p exerted biological functions in OA cell model by targeting HOXA3. Collectively, our findings demonstrated that miR-10a-5p accelerated the progression of OA by targeting HOXA3.

To sum up, the current study revealed that overexpression of miR-10a-5p inhibited chondrocyte proliferation and facilitated chondrocyte apoptosis and cartilage matrix degradation. Furthermore, bioinformatics analyses followed by experimental verification indicated that miR-10a-5p facilitated the progression of OA through triggering the alterations of the whole transcriptome. Our findings shed insight on regulatory mechanism of miR-10a-5p involving in the pathogenesis of OA, which might provide novel therapeutic targets for OA.

## MATERIALS AND METHODS

### Clinical sample collection and animal experiments

Degenerative articular cartilage tissues were obtained from eight OA patients undergoing total hip arthroplasty, while control tissues from seven patients with femoral neck fracture undergoing total hip arthroplasty. Peripheral blood(6ml) from elbow venous blood of another OA patients(n=8) and matched non-OA patients(n=8) were collected for further peripheral blood mononuclear cell (PBMC) isolation. PBMCs were separated using density centrifugation(400×g for 30 min) at room temperature after 6ml of blood was layered onto the same volume of Histopaque-1077 (Sigma-Aldrich). Adult male C57BL/6 mice (n=6; 8 weeks old) from Guangdong Medical Laboratory Animal Center were used to induce DMM OA models as previously described [57]. DMM operations and sham operations were performed in the right knee joints of the mice in DMM group and control group respectively. The left ones of all the mice remained intact. Furthermore, the right knee joints were harvested for subsequent histological analysis. Briefly, knee joints were fixed in 4% paraformaldehyde for 24 h, decalcified in 10% EDTA for 10 days and embedded in paraffin. The medial compartment of the joints was cut into 5 micrometer thick sections and the sections were stained with H&E. All subjects were provided written informed consents before the study. This study was approved by the Human and Animal Experiments Ethics Committee of the Fifth Affiliated Hospital of Sun Yat-Sen University.

### Cell culture and cell transfection

Human primary chondrocytes (HC-a; Catalog #4650) was purchased from ScienCell Research Laboratories. The cells were seeded into 35mm dishes (Corning Incorporated) and maintained in Chondrocyte Medium (ScienCell, Catalog #4651) in a humidified incubator at 37 °C with 5% CO_2_. Only cells within the fifth passage were used for the current experiments. miR-10a-5p mimics and NC were purchased from Guangzhou RiboBio (Guangzhou, China). Si-HOXA3 and their negative controls were purchased from GenePharma (Shanghai, China). The sequences for si-HOXA3 are shown in Supplementary Table 1. Cell transfection was undertaken using Lipofectamine 3000(Invitrogen) according to the protocol of the manufacturer. Cells were harvested for further experiments at 48 hours after transfection.

### Cell viability assay and edu staining

Cell viability was detected using the Cell Counting Kit-8(CCK-8) assay (Dojindo). Cells were seeded into 96-well plates (5000 cells per well) and transfected with miR-10a-5p mimics and mimic-NC or si-NC and si-HOXA3 for 24h, 48h, 72h, and 96h. The OD absorbance at 450 nm was measured with Synergy™ HTX Multi-Mode Microplate Reader (BioTek). Edu staining was performed following the previous protocol [58].

### Flow cytometry assay

Flow cytometry was used to detect the apoptosis rate of HC-a using FITC Annexin V Apoptosis Detection Kit I (BD Pharmingen™) following the protocol of the manufacturer**.** At 48 hours after transfection, cells were washed and collected using pre-chilled phosphate-buffered saline. And then, cells were resuspended in 100 μL of annexin binding buffer(1x) followed by incubating with 5 μL of FITC annexin V and 5 μL of PI working solution at room temperature for 15 min. Subsequently, 400 μL of annexin binding buffer(1x) was added to the mixture. Finally, the apoptosis rate of cells was analyzed by CytoFLEX LX Flow Cytometer (Beckman Coulter).

### RNA isolation, reverse transcription (RT), and real-time quantitative PCR (RT-qPCR)

Total RNA from tissues (articular cartilage) and cells (PBMCs and HC-a) was isolated using HP Total RNA Kit and EZNA Total RNA kit I (Omega Bio-tek, USA) respectively, following the protocols of the manufacturer. The concentration of RNA was measured using NanoDrop 2000 (Thermo Scientific). RNA was synthesized into cDNA using RevertAid First Strand cDNA Synthesis Kit (Thermo Scientific) following the protocols of the manufacturer. PCR reaction was performed with Forget-Me-Not™ EvaGreen® qPCR Master Mix (Biotium) using CFX96^TM^ Real-Time PCR Detection Systems (BIO-RAD) as following: 95°C for 2 min and then 40 cycles of 95°C for 5s, 60°C for 10s, and 72°C for 10s. The relative expression were calculated using the 2^-ΔΔCt^ method. The bulge-loop™ miRNA qRT-PCR Primer Set (one RT primer and a pair of qPCR primers) specific for has-miR-10a-5p is designed and synthetized by RiboBio(Guangzhou, China). The expression of miRNA was normalized to U6, while mRNA, lncRNA, and circRNA normalized to GAPDH. The primer sequences are shown in Supplementary Table 1.

### Western blot

The total protein of cells was extracted using RIPA buffer (Solarbio Biotech, Beijing, China). Protein concentrations were examined using BCA^TM^ protein assay kit (Beyotime Biotechnology, Shanghai, China). A total of 30ug protein was loaded onto the PAGE (EpiZyme, Shanghai, China), separated by electrophoresis and then transferred onto PVDF membranes (Immunoblot, Bio-Rad). The membranes were blocked with 5% non-fat milk (Difco™ Skim Milk, BD) for 1 h at room temperature and incubated with primary antibodies against COL2A1 (Proteintech, Wuhan, China), MMP13(Santa Cruz, UK), BAX(CST, USA), HOXA3(Santa Cruz, USA), GAPDH(CST, USA) at 4 °C overnight. Next, the membranes were incubated with secondary antibodies(Santa Cruz, USA) for 1 h at room temperature. The protein signaling were visualized with ECL chemiluminescence kit(Santa Cruz Biotechnology, Dallas, TX, USA) using Molecular Imager ChemiDoc XRS System(Bio-Rad).

### High throughput sequencing and bioinformatics analysis

The total RNA from HC-a transfected with miR-10a-5p mimics or NC were extracted using the aforementioned methods. The concentration and integrity of total RNA was examined using Qubit 3.0 Fluorometer (Invitrogen, Carlsbad, California) and Agilent 2100 Bioanalyzer (Applied Biosystems, Carlsbad, CA), respectively. High throughput sequencing for the whole transcriptome were performed at Guangzhou Geneseed Biotech Co.,Ltd (Guangzhou, China). Briefly, mRNA, lncRNA and circRNA libraries were established using Total RNA-seq(H/M/R) Library Prep Kit (Illumina) after ribosomal RNA depletion. High throughput sequencing was performed to obtain raw reads on Illumina HiSeq X10 PE150(Illumina, San Diego, CA) after finishing RNA library construction and quality control using Agilent 2100 Bioanalyzer. And then, clean reads were selected after filtering out low-quality data, reads containing jointed-sequence, and reads containing many N sequences from raw reads. Furthermore, effective reads were obtained following removing ribosomal RNA sequences. For mRNAs expression analysis, the reads were mapped to the latest UCSC transcript set using Bowtie 2 version 2.1.0 and the transcripts set from Lncipedia was used for lncRNAs expression analysis [59, 60]. For circRNA expression analysis, the reads were mapped to genome using the STAR and DCC was used to identify the cirRNA and estimate the circRNA expression [61, 62]. For miRNA sequencing, small RNA library was prepared using VAHTSTM Small RNA Library Prep Kit for Illumina^®^(Vazyme Biotech). ExceRpt was used to estimate the miRNA expression in miRBase and novel miRNAs were identified with miRDeep2 [63]. TMM (trimmed mean of M-values) was used to normalize the expression of lncRNA, mRNA, circRNA, and miRNA. Differentially expressed genes were identified using the edgeR program and differentially-expressed genes with adjusted p-value < 0.1 and more than two fold changes were considered to be significant. GO enrichment analyses were visualized using GO plot package, while KEGG enrichment analyses using clusterProfiler package. Protein-protein interaction(PPI) network analysis for differentially expressed mRNAs was performed using String 11.0 and CytoHubba was employed to identify hub genes in the PPI network [64]. Molecular complex detection (MCODE) was used to identify key modules from PPI networks with default algorithms. Network-based gene set enrichment analysis for differentially expressed mRNAs was undertaken using NGSEA [65]. miRanda was used to predict the potential binding relationship between differentially expressed miRNAs and mRNAs, lncRNAs and circRNAs. The circRNA–miRNA–mRNA and lncRNA–miRNA–mRNA regulatory networks were visualized using the Cystoscope software V3.6.0.

### Luciferase reporter assay

To verify the interaction between miR-10a-5p and HOXA3, 293T cells were co-transfected with either the WT or mutated HOXA3 reporter plasmids and miR-10a-5p mimics or miR-NC. After 48 h of incubation, the luciferase signals were measured with a Dual Luciferase Reporter Assay System (Promega).

### Statistical analysis

All the statistical analyses were performed using GraphPad Prism 7.0(GraphPad Software Inc., San Diego, CA). Student T-test was employed to compare the difference across two groups. Results are reported as the mean ± standard deviation (SD). A p value less than 0.05 was considered to be statistically significant.

## Supplementary Material

Supplementary Tables

Supplementary Table 2

Supplementary Table 3

Supplementary Table 6-9

Supplementary Table 10

Supplementary Table 14

Supplementary Table 18

Supplementary Table 19

Supplementary Table 20

## References

[1] Wilson R, Blakely T, Abbott JH. Radiographic knee osteoarthritis impacts multiple dimensions of health-related quality of life: data from the Osteoarthritis Initiative. Rheumatology (Oxford). 2018; 57:891–99. 10.1093/rheumatology/key008 29481663 PMC6251551

[2] Kontio T, Viikari-Juntura E, Solovieva S. Effect of Osteoarthritis on Work Participation and Loss of Working Life-years. J Rheumatol. 2020; 47:597–604. 10.3899/jrheum.181284 31043546

[3] Nüesch E, Dieppe P, Reichenbach S, Williams S, Iff S, Jüni P. All cause and disease specific mortality in patients with knee or hip osteoarthritis: population based cohort study. BMJ. 2011; 342:d1165. 10.1136/bmj.d1165 21385807 PMC3050438

[4] Misra D, Fielding RA, Felson DT, Niu J, Brown C, Nevitt M, Lewis CE, Torner J, Neogi T, and MOST study. Risk of Knee Osteoarthritis With Obesity, Sarcopenic Obesity, and Sarcopenia. Arthritis Rheumatol. 2019; 71:232–37. 10.1002/art.40692 30106249 PMC6374038

[5] Loeser RF, Collins JA, Diekman BO. Ageing and the pathogenesis of osteoarthritis. Nat Rev Rheumatol. 2016; 12:412–20. 10.1038/nrrheum.2016.65 27192932 PMC4938009

[6] Wallace IJ, Worthington S, Felson DT, Jurmain RD, Wren KT, Maijanen H, Woods RJ, Lieberman DE. Knee osteoarthritis has doubled in prevalence since the mid-20th century. Proc Natl Acad Sci USA. 2017; 114:9332–36. 10.1073/pnas.1703856114 28808025 PMC5584421

[7] Deshpande BR, Katz JN, Solomon DH, Yelin EH, Hunter DJ, Messier SP, Suter LG, Losina E. Number of Persons With Symptomatic Knee Osteoarthritis in the US: Impact of Race and Ethnicity, Age, Sex, and Obesity. Arthritis Care Res (Hoboken). 2016; 68:1743–50. 10.1002/acr.22897 27014966 PMC5319385

[8] Gregori D, Giacovelli G, Minto C, Barbetta B, Gualtieri F, Azzolina D, Vaghi P, Rovati LC. Association of Pharmacological Treatments With Long-term Pain Control in Patients With Knee Osteoarthritis: A Systematic Review and Meta-analysis. JAMA. 2018; 320:2564–79. 10.1001/jama.2018.19319 30575881 PMC6583519

[9] Hunter DJ, Bierma-Zeinstra S. Osteoarthritis. Lancet. 2019; 393:1745–59. 10.1016/S0140-6736(19)30417-9 31034380

[10] Valdes AM, Spector TD. Genetic epidemiology of hip and knee osteoarthritis. Nat Rev Rheumatol. 2011; 7:23–32. 10.1038/nrrheum.2010.191 21079645

[11] Beermann J, Piccoli MT, Viereck J, Thum T. Non-coding RNAs in Development and Disease: Background, Mechanisms, and Therapeutic Approaches. Physiol Rev. 2016; 96:1297–325. 10.1152/physrev.00041.2015 27535639

[12] Brandenburger T, Salgado Somoza A, Devaux Y, Lorenzen JM. Noncoding RNAs in acute kidney injury. Kidney Int. 2018; 94:870–81. 10.1016/j.kint.2018.06.033 30348304

[13] Eulalio A, Huntzinger E, Izaurralde E. Getting to the root of miRNA-mediated gene silencing. Cell. 2008; 132:9–14. 10.1016/j.cell.2007.12.024 18191211

[14] Fatemi RP, Velmeshev D, Faghihi MA. De-repressing LncRNA-Targeted Genes to Upregulate Gene Expression: Focus on Small Molecule Therapeutics. Mol Ther Nucleic Acids. 2014; 3:e196. 10.1038/mtna.2014.45 25405465 PMC4461991

[15] Han B, Chao J, Yao H. Circular RNA and its mechanisms in disease: from the bench to the clinic. Pharmacol Ther. 2018; 187:31–44. 10.1016/j.pharmthera.2018.01.010 29406246

[16] Coutinho de Almeida R, Ramos YF, Mahfouz A, den Hollander W, Lakenberg N, Houtman E, van Hoolwerff M, Suchiman HE, Rodríguez Ruiz A, Slagboom PE, Mei H, Kiełbasa SM, Nelissen RG, et al. RNA sequencing data integration reveals an miRNA interactome of osteoarthritis cartilage. Ann Rheum Dis. 2019; 78:270–77. 10.1136/annrheumdis-2018-213882 30504444 PMC6352405

[17] Shen S, Wu Y, Chen J, Xie Z, Huang K, Wang G, Yang Y, Ni W, Chen Z, Shi P, Ma Y, Fan S. CircSERPINE2 protects against osteoarthritis by targeting miR-1271 and ETS-related gene. Ann Rheum Dis. 2019; 78:826–36. 10.1136/annrheumdis-2018-214786 30923232 PMC6579553

[18] Xiao K, Yang Y, Bian Y, Feng B, Li Z, Wu Z, Qiu G, Weng X. Identification of differentially expressed long noncoding RNAs in human knee osteoarthritis. J Cell Biochem. 2019; 120:4620–33. 10.1002/jcb.27750 30302799

[19] Wang H, Zhang H, Sun Q, Wang Y, Yang J, Yang J, Zhang T, Luo S, Wang L, Jiang Y, Zeng C, Cai D, Bai X. Intra-articular Delivery of Antago-miR-483-5p Inhibits Osteoarthritis by Modulating Matrilin 3 and Tissue Inhibitor of Metalloproteinase 2. Mol Ther. 2017; 25:715–27. 10.1016/j.ymthe.2016.12.020 28139355 PMC5363189

[20] Li Y, Li Z, Li C, Zeng Y, Liu Y. Long noncoding RNA TM1P3 is involved in osteoarthritis by mediating chondrocyte extracellular matrix degradation. J Cell Biochem. 2019; 120:12702–12. 10.1002/jcb.28539 30887601

[21] Salmena L, Poliseno L, Tay Y, Kats L, Pandolfi PP. A ceRNA hypothesis: the Rosetta Stone of a hidden RNA language? Cell. 2011; 146:353–58. 10.1016/j.cell.2011.07.014 21802130 PMC3235919

[22] Pan Z, Li GF, Sun ML, Xie L, Liu D, Zhang Q, Yang XX, Xia S, Liu X, Zhou H, Xue ZY, Zhang M, Hao LY, et al. MicroRNA-1224 Splicing CircularRNA-Filip1l in an Ago2-Dependent Manner Regulates Chronic Inflammatory Pain via Targeting Ubr5. J Neurosci. 2019; 39:2125–43. 10.1523/JNEUROSCI.1631-18.2018 30651325 PMC6507086

[23] Ye P, Shi Y, An N, Zhou Q, Guo J, Long X. miR-145 overexpression triggers alteration of the whole transcriptome and inhibits breast cancer development. Biomed Pharmacother. 2018; 100:72–82. 10.1016/j.biopha.2018.01.167 29425746

[24] Hussain N, Zhu W, Jiang C, Xu J, Wu X, Geng M, Hussain S, Cai Y, Xu K, Xu P, Han Y, Sun J, Meng L, Lu S. Down-regulation of miR-10a-5p in synoviocytes contributes to TBX5-controlled joint inflammation. J Cell Mol Med. 2018; 22:241–50. 10.1111/jcmm.13312 28782180 PMC5742673

[25] Hussain N, Zhu W, Jiang C, Xu J, Geng M, Wu X, Hussain S, Wang B, Rajoka MS, Li Y, Tian J, Meng L, Lu S. Down-regulation of miR-10a-5p promotes proliferation and restricts apoptosis via targeting T-box transcription factor 5 in inflamed synoviocytes. Biosci Rep. 2018; 38:38. 10.1042/BSR20180003 29545315 PMC5897746

[26] Vaher H, Runnel T, Urgard E, Aab A, Carreras Badosa G, Maslovskaja J, Abram K, Raam L, Kaldvee B, Annilo T, Tkaczyk ER, Maimets T, Akdis CA, et al. miR-10a-5p is increased in atopic dermatitis and has capacity to inhibit keratinocyte proliferation. Allergy. 2019; 74:2146–56. 10.1111/all.13849 31049964 PMC6817370

[27] Ma Y, Wu Y, Chen J, Huang K, Ji B, Chen Z, Wang Q, Ma J, Shen S, Zhang J. miR-10a-5p Promotes Chondrocyte Apoptosis in Osteoarthritis by Targeting HOXA1. Mol Ther Nucleic Acids. 2019; 14:398–409. 10.1016/j.omtn.2018.12.012 30731321 PMC6365368

[28] Hu Q, Gong W, Gu J, Geng G, Li T, Tian R, Yang Z, Zhang H, Shao L, Liu T, Wan L, Jia J, Yang C, et al. Plasma microRNA Profiles as a Potential Biomarker in Differentiating Adult-Onset Still’s Disease From Sepsis. Front Immunol. 2019; 9:3099. 10.3389/fimmu.2018.03099 30687316 PMC6338094

[29] Guo G, Wang H, Shi X, Ye L, Wu K, Lin K, Ye S, Li B, Zhang H, Lin Q, Ye S, Xue X, Chen C. NovelmiRNA-25 inhibits AMPD2 in peripheral blood mononuclear cells of patients with systemic lupus erythematosus and represents a promising novel biomarker. J Transl Med. 2018; 16:370. 10.1186/s12967-018-1739-5 30577810 PMC6303892

[30] Zhou Y, Zhou B, Pache L, Chang M, Khodabakhshi AH, Tanaseichuk O, Benner C, Chanda SK. Metascape provides a biologist-oriented resource for the analysis of systems-level datasets. Nat Commun. 2019; 10:1523. 10.1038/s41467-019-09234-6 30944313 PMC6447622

[31] Torre D, Lachmann A, Ma’ayan A. BioJupies: Automated Generation of Interactive Notebooks for RNA-Seq Data Analysis in the Cloud. Cell Syst. 2018; 7:556–561.e3. 10.1016/j.cels.2018.10.007 30447998 PMC6265050

[32] Karagkouni D, Paraskevopoulou MD, Chatzopoulos S, Vlachos IS, Tastsoglou S, Kanellos I, Papadimitriou D, Kavakiotis I, Maniou S, Skoufos G, Vergoulis T, Dalamagas T, Hatzigeorgiou AG. DIANA-TarBase v8: a decade-long collection of experimentally supported miRNA-gene interactions. Nucleic Acids Res. 2018; 46:D239–45. 10.1093/nar/gkx1141 29156006 PMC5753203

[33] Shi Y, Hu X, Cheng J, Zhang X, Zhao F, Shi W, Ren B, Yu H, Yang P, Li Z, Liu Q, Liu Z, Duan X, et al. A small molecule promotes cartilage extracellular matrix generation and inhibits osteoarthritis development. Nat Commun. 2019; 10:1914. 10.1038/s41467-019-09839-x 31015473 PMC6478911

[34] Wang XB, Zhao FC, Yi LH, Tang JL, Zhu ZY, Pang Y, Chen YS, Li DY, Guo KJ, Zheng X. MicroRNA-21-5p as a novel therapeutic target for osteoarthritis. Rheumatology (Oxford). 2019. [Epub ahead of print]. 10.1093/rheumatology/kez102 30932160

[35] Flannery CR, Zeng W, Corcoran C, Collins-Racie LA, Chockalingam PS, Hebert T, Mackie SA, McDonagh T, Crawford TK, Tomkinson KN, LaVallie ER, Morris EA. Autocatalytic cleavage of ADAMTS-4 (Aggrecanase-1) reveals multiple glycosaminoglycan-binding sites. J Biol Chem. 2002; 277:42775–80. 10.1074/jbc.M205309200 12202483

[36] Rucci N, Rufo A, Alamanou M, Capulli M, Del Fattore A, Ahrman E, Capece D, Iansante V, Zazzeroni F, Alesse E, Heinegård D, Teti A. The glycosaminoglycan-binding domain of PRELP acts as a cell type-specific NF-kappaB inhibitor that impairs osteoclastogenesis. J Cell Biol. 2009; 187:669–83. 10.1083/jcb.200906014 19951916 PMC2806584

[37] Choi MC, Jo J, Park J, Kang HK, Park Y. NF-κB Signaling Pathways in Osteoarthritic Cartilage Destruction. Cells. 2019; 8:8. 10.3390/cells8070734 31319599 PMC6678954

[38] Monemdjou R, Vasheghani F, Fahmi H, Perez G, Blati M, Taniguchi N, Lotz M, St-Arnaud R, Pelletier JP, Martel-Pelletier J, Beier F, Kapoor M. Association of cartilage-specific deletion of peroxisome proliferator-activated receptor γ with abnormal endochondral ossification and impaired cartilage growth and development in a murine model. Arthritis Rheum. 2012; 64:1551–61. 10.1002/art.33490 22131019 PMC3430604

[39] Vasheghani F, Zhang Y, Li YH, Blati M, Fahmi H, Lussier B, Roughley P, Lagares D, Endisha H, Saffar B, Lajeunesse D, Marshall WK, Rampersaud YR, et al. PPARγ deficiency results in severe, accelerated osteoarthritis associated with aberrant mTOR signalling in the articular cartilage. Ann Rheum Dis. 2015; 74:569–78. 10.1136/annrheumdis-2014-205743 25573665 PMC4345902

[40] Zhu X, Chen F, Lu K, Wei A, Jiang Q, Cao W. PPARγ preservation via promoter demethylation alleviates osteoarthritis in mice. Ann Rheum Dis. 2019; 78:1420–29. 10.1136/annrheumdis-2018-214940 31239244

[41] Lin C, Shao Y, Zeng C, Zhao C, Fang H, Wang L, Pan J, Liu L, Qi W, Feng X, Qiu H, Zhang H, Chen Y, et al. Blocking PI3K/AKT signaling inhibits bone sclerosis in subchondral bone and attenuates post-traumatic osteoarthritis. J Cell Physiol. 2018; 233:6135–47. 10.1002/jcp.26460 29323710

[42] Xie L, Xie H, Chen C, Tao Z, Zhang C, Cai L. Inhibiting the PI3K/AKT/NF-κB signal pathway with nobiletin for attenuating the development of osteoarthritis: in vitro and in vivo studies. Food Funct. 2019; 10:2161–75. 10.1039/C8FO01786G 30938722

[43] Islam N, Haqqi TM, Jepsen KJ, Kraay M, Welter JF, Goldberg VM, Malemud CJ. Hydrostatic pressure induces apoptosis in human chondrocytes from osteoarthritic cartilage through up-regulation of tumor necrosis factor-alpha, inducible nitric oxide synthase, p53, c-myc, and bax-alpha, and suppression of bcl-2. J Cell Biochem. 2002; 87:266–78. 10.1002/jcb.10317 12397608

[44] Hashimoto S, Nishiyama T, Hayashi S, Fujishiro T, Takebe K, Kanzaki N, Kuroda R, Kurosaka M. Role of p53 in human chondrocyte apoptosis in response to shear strain. Arthritis Rheum. 2009; 60:2340–49. 10.1002/art.24706 19644890

[45] Boeuf S, Steck E, Pelttari K, Hennig T, Buneb A, Benz K, Witte D, Sültmann H, Poustka A, Richter W. Subtractive gene expression profiling of articular cartilage and mesenchymal stem cells: serpins as cartilage-relevant differentiation markers. Osteoarthritis Cartilage. 2008; 16:48–60. 10.1016/j.joca.2007.05.008 17604188

[46] Wei T, Kulkarni NH, Zeng QQ, Helvering LM, Lin X, Lawrence F, Hale L, Chambers MG, Lin C, Harvey A, Ma YL, Cain RL, Oskins J, et al. Analysis of early changes in the articular cartilage transcriptisome in the rat meniscal tear model of osteoarthritis: pathway comparisons with the rat anterior cruciate transection model and with human osteoarthritic cartilage. Osteoarthritis Cartilage. 2010; 18:992–1000. 10.1016/j.joca.2010.04.012 20434574

[47] Yoshida K, Suzuki Y, Saito A, Fukuda K, Hamanishi C, Munakata H. Aggrecanase-1 (ADAMTS-4) interacts with alpha1-antitrypsin. Biochim Biophys Acta. 2005; 1725:152–59. 10.1016/j.bbagen.2005.06.009 16099106

[48] Akasaki Y, Reixach N, Matsuzaki T, Alvarez-Garcia O, Olmer M, Iwamoto Y, Buxbaum JN, Lotz MK. Transthyretin deposition in articular cartilage: a novel mechanism in the pathogenesis of osteoarthritis. Arthritis Rheumatol. 2015; 67:2097–107. 10.1002/art.39178 25940564 PMC4519374

[49] Matsuzaki T, Akasaki Y, Olmer M, Alvarez-Garcia O, Reixach N, Buxbaum JN, Lotz MK. Transthyretin deposition promotes progression of osteoarthritis. Aging Cell. 2017; 16:1313–22. 10.1111/acel.12665 28941045 PMC5676063

[50] Yanagisawa A, Ueda M, Sueyoshi T, Nakamura E, Tasaki M, Suenaga G, Motokawa H, Toyoshima R, Kinoshita Y, Misumi Y, Yamashita T, Sakaguchi M, Westermark P, et al. Knee osteoarthritis associated with different kinds of amyloid deposits and the impact of aging on type of amyloid. Amyloid. 2016; 23:26–32. 10.3109/13506129.2015.1115758 26701417

[51] Tsezou A, Iliopoulos D, Malizos KN, Simopoulou T. Impaired expression of genes regulating cholesterol efflux in human osteoarthritic chondrocytes. J Orthop Res. 2010; 28:1033–39. 10.1002/jor.21084 20108316

[52] Tortorella MD, Arner EC, Hills R, Easton A, Korte-Sarfaty J, Fok K, Wittwer AJ, Liu RQ, Malfait AM. Alpha2-macroglobulin is a novel substrate for ADAMTS-4 and ADAMTS-5 and represents an endogenous inhibitor of these enzymes. J Biol Chem. 2004; 279:17554–61. 10.1074/jbc.M313041200 14715656

[53] Luan Y, Kong L, Howell DR, Ilalov K, Fajardo M, Bai XH, Di Cesare PE, Goldring MB, Abramson SB, Liu CJ. Inhibition of ADAMTS-7 and ADAMTS-12 degradation of cartilage oligomeric matrix protein by alpha-2-macroglobulin. Osteoarthritis Cartilage. 2008; 16:1413–20. 10.1016/j.joca.2008.03.017 18485748 PMC2574789

[54] Wang S, Wei X, Zhou J, Zhang J, Li K, Chen Q, Terek R, Fleming BC, Goldring MB, Ehrlich MG, Zhang G, Wei L. Identification of α2-macroglobulin as a master inhibitor of cartilage-degrading factors that attenuates the progression of posttraumatic osteoarthritis. Arthritis Rheumatol. 2014; 66:1843–53. 10.1002/art.38576 24578232 PMC4187342

[55] Xiao M, Li J, Li W, Wang Y, Wu F, Xi Y, Zhang L, Ding C, Luo H, Li Y, Peng L, Zhao L, Peng S, et al. MicroRNAs activate gene transcription epigenetically as an enhancer trigger. RNA Biol. 2017; 14:1326–34. 10.1080/15476286.2015.1112487 26853707 PMC5711461

[56] Pelttari K, Barbero A, Martin I. A potential role of homeobox transcription factors in osteoarthritis. Ann Transl Med. 2015; 3:254. 10.3978/j.issn.2305-5839.2015.09.44 26605300 PMC4620097

[57] Sophocleous A, Huesa C. Osteoarthritis Mouse Model of Destabilization of the Medial Meniscus. Methods Mol Biol. 2019; 1914:281–93. 10.1007/978-1-4939-8997-3_15 30729471

[58] Zhou ZB, Huang GX, Fu Q, Han B, Lu JJ, Chen AM, Zhu L. circRNA.33186 Contributes to the Pathogenesis of Osteoarthritis by Sponging miR-127-5p. Mol Ther. 2019; 27:531–41. 10.1016/j.ymthe.2019.01.006 30692016 PMC6402950

[59] Langmead B, Salzberg SL. Fast gapped-read alignment with Bowtie 2. Nat Methods. 2012; 9:357–59. 10.1038/nmeth.1923 22388286 PMC3322381

[60] Volders PJ, Anckaert J, Verheggen K, Nuytens J, Martens L, Mestdagh P, Vandesompele J. LNCipedia 5: towards a reference set of human long non-coding RNAs. Nucleic Acids Res. 2019; 47:D135–39. 10.1093/nar/gky1031 30371849 PMC6323963

[61] Dobin A, Davis CA, Schlesinger F, Drenkow J, Zaleski C, Jha S, Batut P, Chaisson M, Gingeras TR. STAR: ultrafast universal RNA-seq aligner. Bioinformatics. 2013; 29:15–21. 10.1093/bioinformatics/bts635 23104886 PMC3530905

[62] Cheng J, Metge F, Dieterich C. Specific identification and quantification of circular RNAs from sequencing data. Bioinformatics. 2016; 32:1094–96. 10.1093/bioinformatics/btv656 26556385

[63] Friedländer MR, Mackowiak SD, Li N, Chen W, Rajewsky N. miRDeep2 accurately identifies known and hundreds of novel microRNA genes in seven animal clades. Nucleic Acids Res. 2012; 40:37–52. 10.1093/nar/gkr688 21911355 PMC3245920

[64] Chin CH, Chen SH, Wu HH, Ho CW, Ko MT, Lin CY. cytoHubba: identifying hub objects and sub-networks from complex interactome. BMC Syst Biol. 2014 (Suppl 4); 8:S11. 10.1186/1752-0509-8-S4-S11 25521941 PMC4290687

[65] Han H, Lee S, Lee I. NGSEA: Network-Based Gene Set Enrichment Analysis for Interpreting Gene Expression Phenotypes with Functional Gene Sets. Mol Cells. 2019; 42:579–88. 10.14348/molcells.2019.0065 31307154 PMC6715341

