# Supplementary material for: Overexpression of miR-10a-5p facilitates the progression of osteoarthritis
Source: Aging (Albany NY). 2020 Apr 13;12(7):5948–76. doi: 10.18632/aging.102989 (PMC7185093; doi:10.18632/aging.102989)
Supplement: Supplementary Tables [file aging-12-102989-s007..pdf]

SUPPLEMENTARY TABLES

Supplementary Table 1. Sequences of RT-qPCR primers used.

Gene Name	Forward (5'-3')	Reverse (5'-3')
GAPDH	CCTGCACCACCAACTGCTTA	GGCCATCCACAGTCTTCTGAG
U6	CTCGCTTCGGCAGCACA	AACGCTTCACGAATTTGCGT
OTUD6B-AS	GACATATCCGGGTGACGTTTTT	TTGTTCCACTGTCTTCTGGCATT
has_circ_0010029	AGCTGCTGCTGACTTGAGTG	TTGCTAGTTCTGGAGGCACC
ADM2	TACACGCAGTGCTGGTACG	CTGCTCGTCCAGACATGGC
CAPN6	CAGCAGACTTTTCTGTGATCCA	GGGGACGTTTCCACACCAC
CPXM2	GTGCGCGGGAAGAAATGAC	CCTCCCTTGAGTGATGACACC
HCAR1	AATTTGGCCGTGGCTGATTTC	CCGTAAGGAACACGATGCTCC
NDUFA4L2	CCTGAGCCCCAATGACCAATA	TCTGGCCGGTCCTTCTTCA
SORCS2	GTCACCACCGTCATCGACAAT	TTCGTCTGCGCTGAGGAATAG
CA9	GGATCTACCTACTGTTGAGGCT	CATAGCGCCAATGACTCTGGT
MKX	CGAACACTACCATGATGGGAAA	TTCTGATGACGATGGAGACACTA
NLGN4X	GGTTTACCGCCAATTTGGATACT	CCGTGGGCACGTAGATGTT
SULT1B1	TTTATCTGGCTCGTAATGCCAAG	CCATAGGCCACTTTTCCAGTT
HOXA3	GCAAAAAGCGACCTACTACGA	CGTCGGCGCCCAAAG
COL2A1	TGAGCCATGATTCGCCTCG	CCCTTTGGTCCTGGTTGCC
SOX9	CTGGGAACAACCCGTCTACA	TTCTGGTTGGTCCTCTCTTTCTT
MMP13	CACTTTATGCTTCCTGATGACG	TCCTCGGAGACTGGTAATGG
**si-HOXA3**		
**HOXA3-homo-1**	**CCAACGGGUUCGCUUAUAATT**	**UUAUAAGCGAACCCGUUGGTT**
**HOXA3-homo-2**	**GUGGCUAUCUGAACUCUAUTT**	**AUAGAGUUCAGAUAGCCACTT**
**HOXA3-homo-3**	**CCAGCCCUCUUUGGUCUAATT**	**UUAGACCAAAGAGGGCUGGTT**

Please browse Full Text version to see the data of Supplementary Tables 2 and 3.

Supplementary Table 2. Detailed differentially expressed mRNAs between miR-10a-5p overexpression group(exp) and control group(ctrl).

Supplementary Table 3. Detailed differentially expressed lncRNAs between miR-10a-5p overexpression group(exp) and control group(ctrl).

Supplementary Table 4. Detailed differentially expressed miRNAs between miR-10a-5p overexpression group(exp) and control group(ctrl).

miRNA	Chr_Start_End_Strand	miRBaseID	logFC	foldChange	PValue	pAdj	diffState	M3	M4	NC-3	NC-4
hsa-miR-10a-5p	chr17_48579904_48579926_-	MIMAT0000253	13.23001	9607.938	1.2E-106	4.3E-104	up	404.4072	475.7128	0	0.06014
hsa-miR-100-5p	chr11_122152275_122152296_-	MIMAT0000098	-1.00197	0.499319	1.19E-05	0.001587	down	1604.797	1714.644	2999.09	3574.008
hsa-miR-193b-3p	chr16_14304017_14304038_+	MIMAT0002819	-1.09184	0.469164	1.32E-05	0.001587	down	361.7629	296.0147	452.3804	933.9764
hsa-miR-3619-3p	chr22_46091090_46091111_+	MIMAT0019219	-8.58005	0.002613	0.000192	0.013839	down	0	0	4.471834	7.096537
hsa-miR-496	chr14_101060628_101060649_+	MIMAT0002818	-8.80211	0.00224	0.00012	0.010782	down	0	0	5.438717	8.058779

Supplementary Table 5. Detailed differentially expressed circRNAs between miR-10a-5p overexpression group(exp) and control group(ctrl).

Chr_Start_End_Strand	circBankID	circbaseID	splicedSeqLength	Gene	mouse_conserved_circRNA	startEndRegion	logFC	foldChange	PValue	pAdj	diffState	M1	M2	NC-3	NC-4
chr17_81042813_81043199_+				METRNL		exon-exon	6.462089	88.16225	2.88E-05	0.003701	up	1.04258	0.227358	0	0
chr14_99723807_99724176_-	hsa_circBCL11B_002	hsa_circ_0033144	369	BCL11B	mmu_circ_0000414	exon-exon	6.010497	64.46734	8.65E-06	0.001669	up	0.417032	0.530503	0	0
chr15_89656955_89659752_+	hsa_circABHD2_007	hsa_circ_0007099	300	ABHD2	mmu_circ_0014410	exon-exon	6.0028	64.12433	7.12E-05	0.005499	up	0.695053	0.227358	0	0
chr13_76195898_76335174_+				LMO7		exon-exon	5.322277	40.00966	0.000288	0.015907	up	0.347527	0.227358	0	0
chr7_26232870_26232993_-	hsa_circHNRNPA2B1_019	hsa_circ_0001689	123	HNRNPA2B1		exon-exon	5.322277	40.00966	0.000288	0.015907	up	0.347527	0.227358	0	0
chr14_35331249_35331528_-	hsa_circBAZ1A_004	hsa_circ_0006137	279	BAZ1A		exon-exon	4.920285	30.27982	0.001735	0.076806	up	0.208516	0.227358	0	0
chr16_85667519_85667738_+	hsa_circKIAA0182_001	hsa_circ_0000722	219	GSE1	mmu_circ_0001730	exon-exon	4.110917	17.27864	6.68E-05	0.005499	up	0.417032	1.591509	0	0.090727
chr1_155646338_155649303_-	hsa_circYY1AP1_008	hsa_circ_0014606	459	YY1AP1		exon-exon	2.450128	5.464644	0.002827	0.099192	up	0.973075	0.454717	0.160478	0.090727
chr5_64747301_64769779_-	hsa_circADAMTS6_004	hsa_circ_0072688	1352	ADAMTS6	mmu_circ_0004419	exon-exon	-2.12968	0.228508	0.001968	0.076806	down	0.139011	0.303145	0.802389	1.08872
chr1_14104912_14109326_+	hsa_circPRDM2_011	hsa_circ_0010029	4414	PRDM2		exon-exon	-5.25035	0.026272	0.00199	0.076806	down	0	0	0.160478	0.362907

Please browse Full Text version to see the data of Supplementary Tables 6 to 10.

Supplementary Table 6. Detailed GO analysis (biological process) of differentially expressed mRNAs.

Supplementary Table 7. Detailed GO analysis (cellular component) of differentially expressed mRNAs.

Supplementary Table 8. Detailed GO analysis(molecular function) of differentially expressed mRNAs.

Supplementary Table 9. Detailed KEGG analysis of differentially expressed mRNAs.

Supplementary Table 10. Detailed GO analysis(biological process) of ci-genes of differentially expressed lncRNAs.

Supplementary Table 11. Detailed GO analysis (cellular component) of ci-genes of differentially expressed lncRNAs.

databaseID	Description	type	geneRatio	bgRatio	pvalue	padj	qvalue	enrichScore	overlapGeneList	overlapGeneCount
GO:0030120	vesicle coat	cellular component	5/267	48/18678	0.000596	0.177015	0.177015	7.286985	PEF1/COPZ1/EPN2/SYNRG/SEC24B	5
GO:0030117	membrane coat	cellular component	6/267	86/18678	0.001459	0.177015	0.177015	4.880585	PEF1/COPZ1/EPN2/SYNRG/VPS33B/SEC24B	6
GO:0048475	coated membrane	cellular component	6/267	86/18678	0.001459	0.177015	0.177015	4.880585	PEF1/COPZ1/EPN2/SYNRG/VPS33B/SEC24B	6
GO:0031463	Cul3-RING ubiquitin ligase complex	cellular component	3/267	34/18678	0.012465	0.640758	0.640758	6.172505	ENC1/GAN/PEF1	3
GO:0005667	transcription factor complex	cellular component	9/267	270/18678	0.015966	0.640758	0.640758	2.331835	BRF1/BRF2/JUNB/SFPQ/SNF8/HAND2/SIX1/TBX2/NFYC	9
GO:0030658	transport vesicle membrane	cellular component	7/267	188/18678	0.018699	0.640758	0.640758	2.60471	PEF1/RAB1A/SCAMP1/DNM1L/SPRED2/SYNRG/SEC24B	7
GO:0030118	clathrin coat	cellular component	3/267	41/18678	0.020646	0.640758	0.640758	5.118663	EPN2/SYNRG/VPS33B	3
GO:0044815	DNA packaging complex	cellular component	5/267	113/18678	0.023175	0.640758	0.640758	3.095356	HIST2H3D/SMC4/H2BFWT/HIST1H2AI/HIST4H4	5
GO:0062023	collagen-containing extracellular matrix	cellular component	11/267	399/18678	0.02902	0.640758	0.640758	1.928586	A1BG/ADAMTS1/COL4A5/COL9A2/FGA/FMOD/SERPINB6/LOXL1/ADAMTS9/IGFBP7/LTBP3	11
GO:0030135	coated vesicle	cellular component	8/267	261/18678	0.034503	0.640758	0.640758	2.144216	EPGN/PEF1/SCAMP1/COPZ1/EPN2/SYNRG/VPS33B/SEC24B	8
GO:0030662	coated vesicle membrane	cellular component	6/267	169/18678	0.034792	0.640758	0.640758	2.483611	EPGN/PEF1/COPZ1/EPN2/SYNRG/SEC24B	6
GO:0031012	extracellular matrix	cellular component	12/267	468/18678	0.037407	0.640758	0.640758	1.793719	A1BG/ADAMTS1/CHI3L1/COL4A5/COL9A2/FGA/FMOD/SERPINB6/LOXL1/ADAMTS9/IGFBP7/LTBP3	12

Supplementary Table 12. Detailed GO analysis(molecular function) of ci-genes of differentially expressed lncRNAs.

databaseID	Description	type	geneRatio	bgRatio	pvalue	padj	qvalue	enrichScore	overlapGeneList	Overlap GeneCount
GO:0016651	oxidoreductase activity, acting on NAD(P)H	molecular function	6/247	96/16969	0.002787	0.375998	0.372662	4.293775	AKR1C2/AKR1C3/CBR1/TXNRD2/DHRS4/MIOX	6
GO:0004033	aldo-keto reductase (NADP) activity	molecular function	3/247	23/16969	0.004351	0.375998	0.372662	8.960922	AKR1C2/AKR1C3/MIOX	3
GO:0004722	protein serine/threonine phosphatase activity	molecular function	4/247	49/16969	0.005557	0.375998	0.372662	5.608196	CTDSP2/DUSP4/PPA2/UBLCP1	4
GO:0003755	peptidyl-prolyl cis-trans isomerase activity	molecular function	3/247	26/16969	0.006186	0.375998	0.372662	7.92697	FKBP11/FKBP2/FKBP8	3
GO:0016859	cis-trans isomerase activity	molecular function	3/247	28/16969	0.007629	0.375998	0.372662	7.360758	FKBP11/FKBP2/FKBP8	3
GO:0016614	oxidoreductase activity, acting on CH-OH group of donors	molecular function	6/247	120/16969	0.008242	0.375998	0.372662	3.43502	AKR1C2/AKR1C3/CBR1/DHRS4/MIOX/VKORC1L1	6
GO:0016655	oxidoreductase activity, acting on NAD(P)H, quinone or similar compound as acceptor	molecular function	4/247	57/16969	0.009467	0.375998	0.372662	4.821081	AKR1C2/AKR1C3/CBR1/DHRS4	4
GO:0097718	disordered domain specific binding	molecular function	3/247	31/16969	0.010139	0.375998	0.372662	6.648426	FKBP8/KCNA1/GAPDH	3
GO:0046982	protein heterodimerization activity	molecular function	14/247	497/16969	0.014367	0.480885	0.476619	1.935223	GABPB1/APOA2/BCL2/HIST2H3D/PEF1/SMC4/SRGAP2C/TMCC1/HAND2/H2BFWT/HIST1H2AI/HIST4H4/NFYC/SUCLG2	14
GO:0019902	phosphatase binding	molecular function	7/247	177/16969	0.015129	0.480885	0.476619	2.716965	BCL2/EIF2AK3/PPP1R3G/SYK/JAK1/VCP/MAGI2	7
GO:0004715	non-membrane spanning protein tyrosine kinase activity	molecular function	3/247	37/16969	0.016446	0.487889	0.483561	5.570303	CLK1/SYK/JAK1	3
GO:0016706	oxidoreductase activity, acting on paired donors, with incorporation or reduction of molecular oxygen, 2-oxoglutarate as one donor, and incorporation of one atom each of oxygen into both donors	molecular function	3/247	40/16969	0.020259	0.514465	0.509901	5.15253	ASPHD1/EGLN1/KDM4C	3
GO:0016616	oxidoreductase activity, acting on the CH-OH group of donors, NAD or NADP as acceptor	molecular function	5/247	108/16969	0.02084	0.514465	0.509901	3.180574	AKR1C2/AKR1C3/CBR1/DHRS4/MIOX	5
GO:0005201	extracellular matrix structural constituent	molecular function	6/247	158/16969	0.028244	0.514465	0.509901	2.608876	CHI3L1/COL4A5/COL9A2/FGA/FMOD/IGFBP7	6
GO:0016853	isomerase activity	molecular function	5/247	122/16969	0.03299	0.514465	0.509901	2.81559	FKBP11/FKBP2/FKBP8/HPGDS/K	5

									ATNAL1	
GO:0070888	E-box binding	molecular function	3/247	49/16969	0.034364	0.514465	0.509901	4.206147	SFPQ/HAND2/TFAP4	3
GO:0019903	protein phosphatase binding	molecular function	5/247	132/16969	0.043906	0.514465	0.509901	2.602288	BCL2/EIF2AK3/PPP1R3G/JAK1/VCP	5
GO:0015171	amino acid transmembrane transporter activity	molecular function	3/247	55/16969	0.045956	0.514465	0.509901	3.747295	SLC25A22/SLC36A1/SLC6A18	3

Supplementary Table 13. Detailed KEGG analysis of ci-genes of differentially expressed lncRNAs.

databaseID	Description	geneRatio	bgRatio	pvalue	padj	qvalue	enrichScore	overlapGeneList	overlapGene Count
hsa04724	Glutamatergic synapse	5/102	114/7946	0.015404	0.823484	0.823484	3.416753	GNAI1/GNG11/GRM6/DLGAP1/SHANK2	5
hsa05034	Alcoholism	6/102	184/7946	0.030703	0.823484	0.823484	2.540281	GNAI1/GNG11/HIST2H3D/H2BFWT/HIST1H2AI/HIST4H4	6
hsa05202	Transcriptional misregulation in cancer	6/102	186/7946	0.032134	0.823484	0.823484	2.512966	HIST2H3D/IGFBP3/TGFBR2/ID2/SIX1/ZBTB16	6
hsa04974	Protein digestion and absorption	4/102	95/7946	0.033515	0.823484	0.823484	3.280083	COL4A5/COL9A2/SLC36A1/SLC7A7	4
hsa00240	Pyrimidine metabolism	3/102	57/7946	0.036469	0.823484	0.823484	4.100103	DCTD/NT5E/NT5M	3
hsa05134	Legionellosis	3/102	57/7946	0.036469	0.823484	0.823484	4.100103	CLK1/RAB1A/VCP	3
hsa00590	Arachidonic acid metabolism	3/102	63/7946	0.046869	0.823484	0.823484	3.709617	AKR1C3/CBR1/HPGDS	3

Please browse Full Text version to see the data of Supplementary Table 14.

Supplementary Table 14. Detailed GO analysis (biological process) of parent genes of differentially expressed circRNAs.

Supplementary Table 15. Detailed GO analysis(cellular component) of parent genes of differentially expressed circRNAs.

Database ID	Description	type	geneRatio	bgRatio	pvalue	padj	qvalue	enrichScore	Overlap GeneList	Overlap GeneCount
GO:0070603	SWI/SNF superfamily-type complex	cellular component	2/9	68/18678	0.000462	0.013516	0.007114	61.03922	YY1AP1/BAZ1A	2
GO:1904949	ATPase complex	cellular component	2/9	92/18678	0.000845	0.013516	0.007114	45.11594	YY1AP1/BAZ1A	2
GO:0097524	sperm plasma membrane	cellular component	1/9	6/18678	0.002888	0.030805	0.016213	345.8889	ABHD2	1
GO:0031010	ISWI-type complex	cellular component	1/9	11/18678	0.005289	0.038433	0.020228	188.6667	BAZ1A	1
GO:0031011	Ino80 complex	cellular component	1/9	15/18678	0.007206	0.038433	0.020228	138.3556	YY1AP1	1
GO:0033202	DNA helicase complex	cellular component	1/9	15/18678	0.007206	0.038433	0.020228	138.3556	YY1AP1	1
GO:0000790	nuclear chromatin	cellular component	2/9	309/18678	0.009095	0.041578	0.021883	13.43258	YY1AP1/BAZ1A	2
GO:0097346	INO80-type complex	cellular component	1/9	22/18678	0.010553	0.042213	0.022217	94.33333	YY1AP1	1
GO:0044454	nuclear chromosome part	cellular component	2/9	469/18678	0.020151	0.071649	0.03771	8.850036	YY1AP1/BAZ1A	2
GO:0000785	chromatin	cellular component	2/9	500/18678	0.022729	0.072732	0.03828	8.301333	YY1AP1/BAZ1A	2
GO:0015030	Cajal body	cellular component	1/9	75/18678	0.035571	0.096195	0.050629	27.67111	HNRNPA2B1	1
GO:0071013	catalytic step 2 spliceosome	cellular component	1/9	82/18678	0.038833	0.096195	0.050629	25.30894	HNRNPA2B1	1
GO:0036126	sperm flagellum	cellular component	1/9	84/18678	0.039763	0.096195	0.050629	24.70635	ABHD2	1
GO:0097729	9+2 motile cilium	cellular component	1/9	89/18678	0.042085	0.096195	0.050629	23.31835	ABHD2	1
GO:0001669	acrosomal vesicle	cellular component	1/9	103/18678	0.04856	0.099885	0.052571	20.14887	ABHD2	1
GO:0016363	nuclear matrix	cellular component	1/9	106/18678	0.049942	0.099885	0.052571	19.57862	HNRNPA2B1	1

Supplementary Table 16. Detailed GO analysis (molecular function) of parent genes of differentially expressed circRNAs.

Database ID	Description	type	geneRatio	bgRatio	pvalue	padj	qvalue	enrichScore	Overlap GeneList	overlapGene Count
GO:0097157	pre-mRNA intronic binding	molecular function	1/10	6/16969	0.003531	0.047093	0.022776	282.8167	HNRNPA2B1	1
GO:0047372	acylglycerol lipase activity	molecular function	1/10	7/16969	0.004119	0.047093	0.022776	242.4143	ABHD2	1
GO:1990247	N6-methyladenosine-containing RNA binding	molecular function	1/10	7/16969	0.004119	0.047093	0.022776	242.4143	HNRNPA2B1	1
GO:0098505	G-rich strand telomeric DNA binding	molecular function	1/10	10/16969	0.005879	0.047093	0.022776	169.69	HNRNPA2B1	1
GO:0043047	single-stranded telomeric DNA binding	molecular function	1/10	11/16969	0.006465	0.047093	0.022776	154.2636	HNRNPA2B1	1
GO:0098847	sequence-specific single stranded DNA binding	molecular function	1/10	13/16969	0.007637	0.047093	0.022776	130.5308	HNRNPA2B1	1

GO:0035198	miRNA binding	molecular function	1/10	31/16969	0.018124	0.077857	0.037655	54.73871	HNRNPA2B1	1
GO:0042162	telomeric DNA binding	molecular function	1/10	34/16969	0.019862	0.077857	0.037655	49.90882	HNRNPA2B1	1
GO:0001228	DNA-binding transcription activator activity, RNA polymerase II-specific	molecular function	2/10	390/16969	0.020986	0.077857	0.037655	8.702051	BCL11B/PRDM2	2
GO:0061980	regulatory RNA binding	molecular function	1/10	37/16969	0.021597	0.077857	0.037655	45.86216	HNRNPA2B1	1
GO:0018024	histone-lysine N-methyltransferase activity	molecular function	1/10	43/16969	0.02506	0.077857	0.037655	39.46279	PRDM2	1
GO:0042054	histone methyltransferase activity	molecular function	1/10	52/16969	0.030233	0.077857	0.037655	32.63269	PRDM2	1
GO:0003707	steroid hormone receptor activity	molecular function	1/10	54/16969	0.031379	0.077857	0.037655	31.42407	ABHD2	1
GO:0036002	pre-mRNA binding	molecular function	1/10	54/16969	0.031379	0.077857	0.037655	31.42407	HNRNPA2B1	1
GO:0016279	protein-lysine N-methyltransferase activity	molecular function	1/10	57/16969	0.033096	0.077857	0.037655	29.77018	PRDM2	1
GO:0016278	lysine N-methyltransferase activity	molecular function	1/10	58/16969	0.033668	0.077857	0.037655	29.2569	PRDM2	1
GO:0003730	mRNA 3'-UTR binding	molecular function	1/10	67/16969	0.0388	0.084446	0.040842	25.32687	HNRNPA2B1	1
GO:0008276	protein methyltransferase activity	molecular function	1/10	80/16969	0.046169	0.087124	0.042137	21.21125	PRDM2	1
GO:0042562	hormone binding	molecular function	1/10	81/16969	0.046734	0.087124	0.042137	20.94938	ABHD2	1
GO:0008170	N-methyltransferase activity	molecular function	1/10	86/16969	0.049553	0.087124	0.042137	19.7314	PRDM2	1

Supplementary Table 17. Detailed KEGG analysis of parent genes of differentially expressed circRNAs.

databaseID	Description	geneRatio	bgRatio	pvalue	padj	qvalue	enrichScore	overlapGeneList	overlapGeneCount
hsa00310	Lysine degradation	1/3	61/7946	0.022857	0.039856	0.013984	43.42077	PRDM2	1
hsa04520	Adherens junction	1/3	71/7946	0.02657	0.039856	0.013984	37.30516	LMO7	1

Please browse Full Text version to see the data of Supplementary Tables 18 to 20.

Supplementary Table 18. Detailed GSEA(biological process) of differentially expressed mRNAs.

Supplementary Table 19. Detailed GSEA(KEGG) of differentially expressed mRNAs.

Supplementary Table 20. The network degreee analysis of all the differentially-expressed mRNAs using CytoHubba.
