# Supplementary figures and images for: Overexpression of miR-10a-5p facilitates the progression of osteoarthritis
Source: Aging (Albany NY). 2020 Apr 13;12(7):5948–76. doi: 10.18632/aging.102989 (PMC7185093; doi:10.18632/aging.102989)

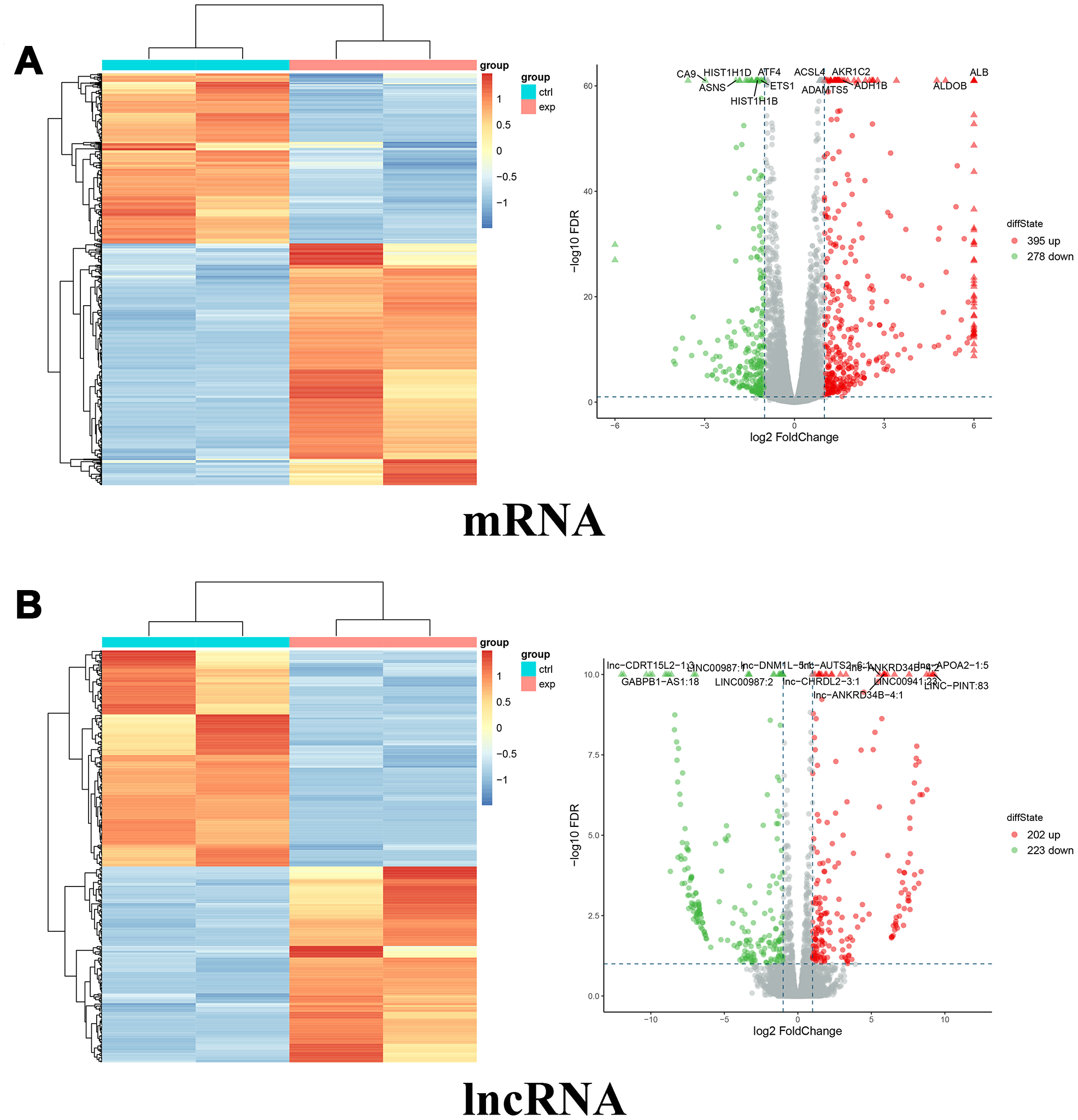

Supplement: Supplementary Table 6-9 [file aging-12-102989-s009..tif]

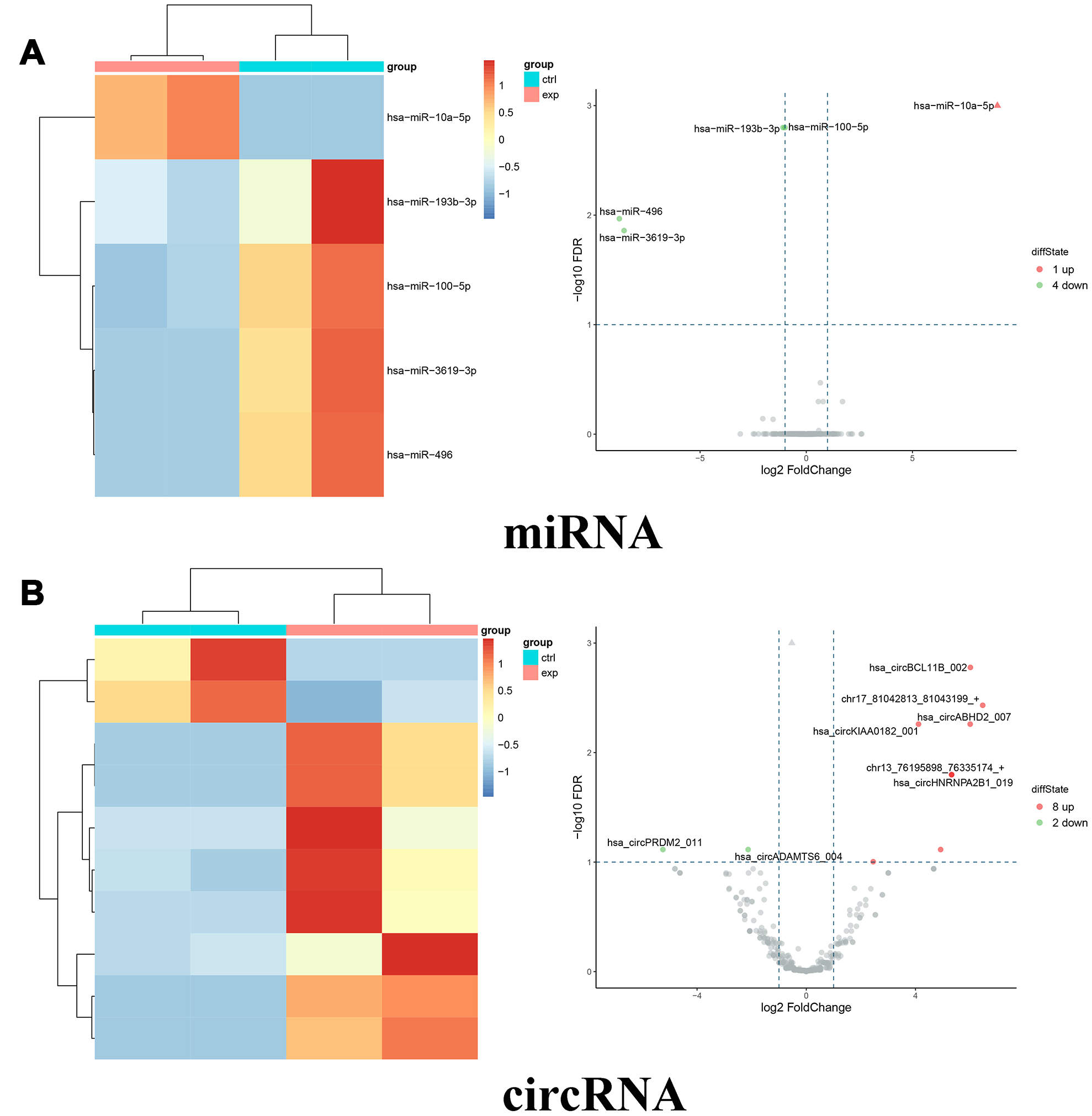

Supplement: Supplementary Table 10 [file aging-12-102989-s008..tif]
